# Key candidate genes and pathways in T lymphoblastic leukemia/lymphoma identified by bioinformatics and serological analyses

**DOI:** 10.3389/fimmu.2024.1341255

**Published:** 2024-02-23

**Authors:** Yansong Ren, Haoyue Liang, Yali Huang, Yuyang Miao, Ruihua Li, Junlian Qiang, Lihong Wu, Jinfeng Qi, Ying Li, Yonghui Xia, Lunhui Huang, Shoulei Wang, Xiaodong Kong, Yuan Zhou, Qiang Zhang, Guoqing Zhu

**Affiliations:** ^1^State Key Laboratory of Experimental Hematology, National Clinical Research Center for Blood Diseases, Haihe Laboratory of Cell Ecosystem, Institute of Hematology & Blood Diseases Hospital, Chinese Academy of Medical Sciences & Peking Union Medical College, Tianjin, China; ^2^Tianjin Institutes of Health Science, Tianjin, China; ^3^Clinical Laboratory of Zhengning County People's Hospital, Qingyang, Gansu, China; ^4^Department of Geriatrics, Tianjin Medical University General Hospital, Tianjin Geriatrics Institute, Tianjin, China

**Keywords:** T-ALL, T-LBL, bioinformatics analysis, serology, protein-protein interaction networks

## Abstract

T-cell acute lymphoblastic leukemia (T*-*ALL)/T-cell lymphoblastic lymphoma (T-LBL) is an uncommon but highly aggressive hematological malignancy. It has high recurrence and mortality rates and is challenging to treat. This study conducted bioinformatics analyses, compared genetic expression profiles of healthy controls with patients having T-ALL/T-LBL, and verified the results through serological indicators. Data were acquired from the GSE48558 dataset from Gene Expression Omnibus (GEO). T-ALL patients and normal T cells-related differentially expressed genes (DEGs) were investigated using the online analysis tool GEO2R in GEO, identifying 78 upregulated and 130 downregulated genes. Gene Ontology (GO) and protein-protein interaction (PPI) network analyses of the top 10 DEGs showed enrichment in pathways linked to abnormal mitotic cell cycles, chromosomal instability, dysfunction of inflammatory mediators, and functional defects in T-cells, natural killer (NK) cells, and immune checkpoints. The DEGs were then validated by examining blood indices in samples obtained from patients, comparing the T-ALL/T-LBL group with the control group. Significant differences were observed in the levels of various blood components between T-ALL and T-LBL patients. These components include neutrophils, lymphocyte percentage, hemoglobin (HGB), total protein, globulin, erythropoietin (EPO) levels, thrombin time (TT), D-dimer (DD), and C-reactive protein (CRP). Additionally, there were significant differences in peripheral blood leukocyte count, absolute lymphocyte count, creatinine, cholesterol, low-density lipoprotein, folate, and thrombin times. The genes and pathways associated with T-LBL/T-ALL were identified, and peripheral blood HGB, EPO, TT, DD, and CRP were key molecular markers. This will assist the diagnosis of T-ALL/T-LBL, with applications for differential diagnosis, treatment, and prognosis.

## 1 Introduction

T-cell acute lymphoblastic leukemia (T*-*ALL)/T-cell lymphoblastic lymphoma (T-LBL) is a relatively rare and highly malignant lymphoproliferative disease in the T-cell line of lymphoblastic tumors. The majority of cases arise from lymph nodes or extranodal tissues and are classified as T-LBL, but a lesser proportion of cases originating from the peripheral blood and bone marrow are referred to as T-ALL. Both forms have similar clinical and laboratory characteristics, including cellular morphology, immune phenotype, genotype, cytogenetics, clinical manifestations, and prognosis. T-LBL is clinically diagnosed when there is an absence of malignant cell infiltration in the peripheral blood or bone marrow or when less than 25% of tumor lymphoblasts appear in the bone marrow. On the contrary, T-ALL is identified when there is blood and bone marrow infiltration and when the levels of lymphoblasts in the bone marrow surpass 25% (1, 2).

T-LBL is much more common than B-lymphoblastic lymphoma, with 85% -90% of T-LBL cases belonging to the T-cell lineage (3). T-LBL often presents with mediastinal masses, followed by invasion of the peripheral lymph nodes, liver, spleen, skin, pharyngeal lymph nodes, CNS, or gonads (4). T-ALL has a high degree of invasiveness and primarily affects adolescents, with a higher prevalence in males, constituting 25% of all cases of ALL in adults. Large masses and white blood cell lesions in the mediastinum or other areas, together with relatively reduced bone marrow hematopoiesis, are the typical presentation of T-ALL (3). T-ALL/T-LBL is highly invasive and is associated with high levels of recurrence, poor long-term survival, and unfavorable prognosis. Hence, relevant bioinformatics and serological research is urgently needed.

Microarray and sequencing data are combined in the Gene Expression Omnibus (GEO, http://www.ncbi.nlm.nih.gov/geo/), which has greatly advanced the understanding of cancer. GEO incorporates data from independent studies and clinical samples. Comparative analysis is limited by its inability to combine data from several independent sources because of variations in research approaches and microarray platforms. Bioinformatics analysis is effective for the large-scale evaluation of high-throughput data across platforms (5).

Here, the microarray and RNA-seq data were used from the TCGA to identify differentially expressed genes (DEGs) that could then be investigated *via* protein-protein interaction (PPI) network and Gene Ontology (GO) enrichment analyses. The levels of these genes were then verified using serological indicators in patient samples to provide a reference for diagnosing and treating T-ALL/T-LBL. Due to the differences in prognostic factors and treatment strategies between T-ALL and T-LBL, it may be necessary to evaluate related gene abnormalities through candidate genes or large-scale unbiased methods as treatment becomes more targeted. The objectives of this research were to understand the pathogenesis of T-ALL/T-LBL better, identify novel therapeutic targets, aid in developing medications with increased potency, less toxicity, and more focused effects, increase the cure rate, and alleviate pain and side effects.

## 2 Materials and methods

### 2.1 Population

**Figure 1:**
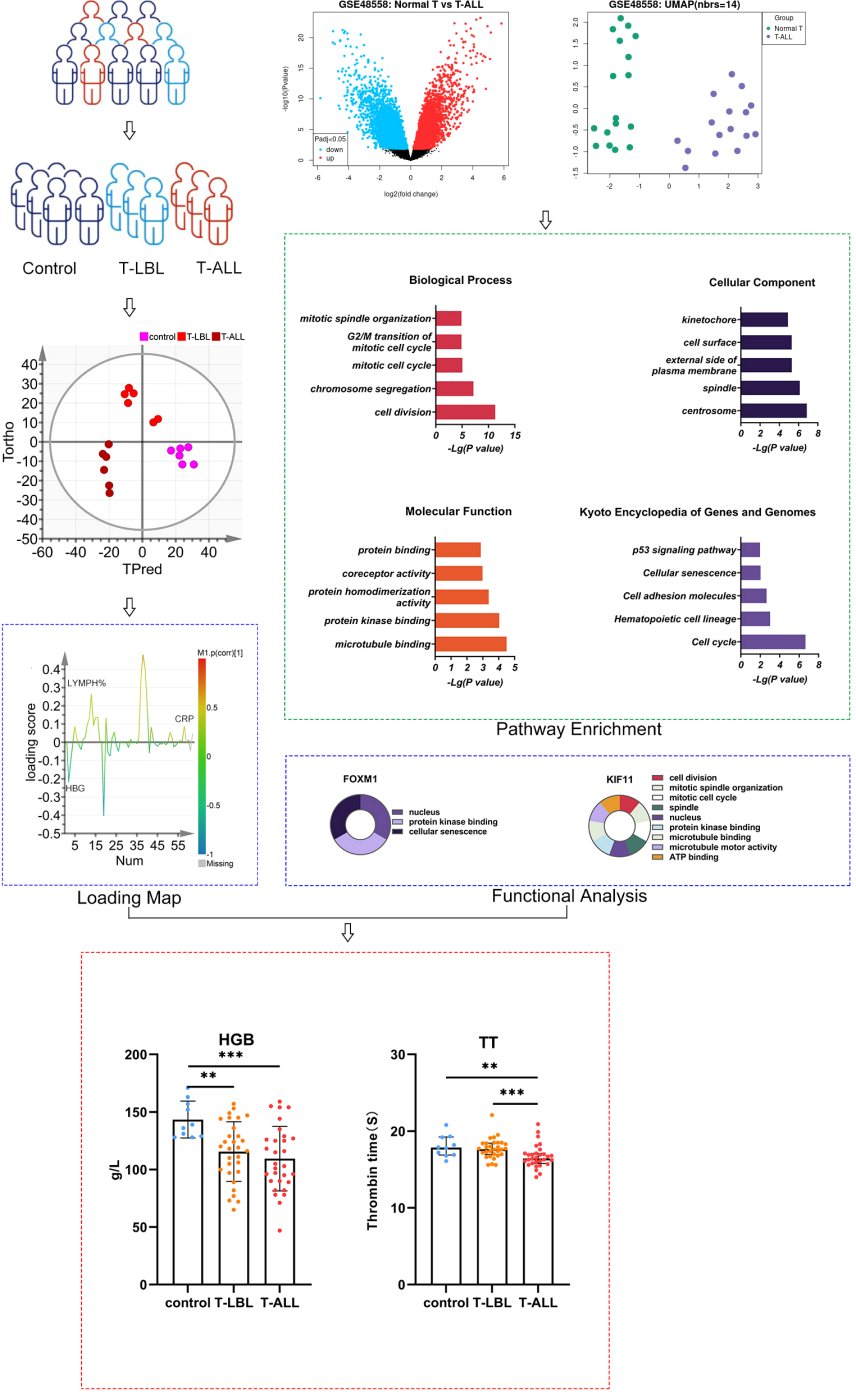
Schematic of the study design. **P ≤ 0.01, ***p = 0.001

The study participants were recruited from the Hematology Hospital of the Chinese Academy of Medical Sciences (Institute of Hematology, Chinese Academy of Medical Sciences) after obtaining consent from the Ethics Committee (KT2020016-EC-2) to perform this experiment. Moreover, biochemical analysis data were obtained from the clinical testing center at the same institution. Cumulatively, 91 participants were allocated to different groups according to their diagnosis, namely, healthy controls (n = 31, including 12 males, aged 32–72 years), the T-LBL group (n = 30, including 21 males, aged 4–82 years) and the T-ALL group (n = 30, including 23 males, aged 6–58 years; **Figure 1**; **Supplementary Tables 1-4**). All participants underwent examinations, including hematological, bone marrow, and cytogenetic assessments and genotype and immunological phenotype analysis. Diagnoses were confirmed by experienced hematologists.

### 2.2 Collection of general clinical data

Data on the medical histories, clinical characteristics, and blood markers were gathered from the study participants. After fasting for 10 h, the serum was collected, and peripheral blood biochemical indicators were measured using a fully automated biochemical analyzer. These indicators included hemoglobin level (HGB), platelet (PLT) and white blood cell (WBC) counts, peripheral blood neutrophil and lymphocyte absolute values and percentages (NEU# and NEU%, and LYMPH# and LYMPH%, respectively), total protein (TP), globulin (GLB), phosphorus (P), albumin (ALB), alkaline phosphatase (ALP), total and direct bilirubin (TBIL and DBIL), urea (URA), creatinine (CREA), lactate dehydrogenase (LDH), α-hydroxybutyrate dehydrogenase (α-HBDH), uric acid (UA), creatine kinase and its isoenzyme (CK and CK-MB), total bile acid (TBA), amylase (AMY), sodium (Na), potassium (K), magnesium (Mg), carbon dioxide-combining power (CO_2_CP), glucose (GLU), triglycerides (TG), total cholesterol (TC), high-density lipoprotein cholesterol (HDL), low-density lipoprotein cholesterol (LDL), folate (FA), vitamin B12 (B12), aspartate aminotransferase (AST), ferritin (F), erythropoietin (EPO), iron (Fe), chlorine (Cl), unsaturated iron-binding capacity (UIBC), total iron-binding capacity (TIBC), iron saturation (ISAT), glutamyl transpeptidase (GGT), prothrombin time (PT), international standardization ratio (INR), partial thromboplastin time (APTT), thrombin time (TT), fibrinogen (FIB), antithrombin III (ATIII), fibrinogen decomposition products (FDB), D-dimer (DD), alanine aminotransferase (ALT), immunoglobulin G (IgG), immunoglobulin A (IgA), immunoglobulin M (IgM), complement C3 (C3) complement C4 (C4), C-reactive protein (CRP), rheumatoid factor, anti-streptolysin O (ASO), and calcium (Ca).

### 2.3 OPLS-DA multivariate statistical method to establish identification model

Orthogonal partial least squares discriminant analysis (OPLS-DA) was performed using SIMCA 14.1 to compare indicator levels between the control, T-LBL, and T-ALL groups. The model performance was examined using the R^2^ and Q^2^ goodness-of-fit parameters. The null hypothesis posits 200 resamplings of the model using random alterations in the y-matrix. Model reliability was assessed using receiver operating characteristic (ROC) curves. V + S was utilized to evaluate the potential of biomarkers for the identified indicators. Potential biomarkers were selected from parameters with Variable Importance (VIP) > 1.0 and top-five p (cor) ranking, using correlation coefficients and distance from the center in the V + S plot. Potential biomarkers showing significance levels of *p* < 0.05 were identified.

### 2.4 Identification of DEGs in the T-ALL and control groups

#### 2.4.1 Data sources

The *Homo sapiens* dataset GSE48558 from GEO was utilized in the present study. The dataset included 12 gene expression profile samples, all obtained using the GPL6244 Affymetrix Human Gene 1.0 ST Array platform.

#### 2.4.2 DEG identification

DEG identification was done using the GEO2R online tool in GEO, which uses the GEOquery and limma packages in R. The screening criteria for DEGs were *p* < 0.05 and | logFC | > 3.

#### 2.4.3 Functional analysis of DEGs

DAVID (https://david.ncifcrf.gov/) was used for gene ontology (GO) and Kyoto Encyclopedia of Genes and Genomes (KEGG) analyses using *p* < 0.05 as the threshold.

#### 2.4.4 PPI networks and identification of key genes

PPI networks of the DEGs were generated with STRING (Version: 10.0; http://www.string-db.org/) with a PPI score threshold of 0.4, indicating medium confidence. The resulting networks were visualized using Cytoscape version 3.6.0. The network nodes were scored, and those scoring in the top 10 regarding expression level were designated as key nodes.

### 2.5 Statistical analysis

Statistical Package for the Social Sciences (SPSS; Version: 26) was used to analyze the OPLS-DA data. Normally distributed data are represented by mean ± standard deviation (SD; x ± s) and analyzed by one-way analysis of variance (ANOVA). Pairwise comparisons between groups with homogeneous variance were done using the LSD method, while pairwise comparisons between groups with heterogeneous variance were done using Tamhane’s T2 method. Non-normally distributed data are represented by medians (quartile range) and analyzed using Kruskal Wallis rank-sum tests. A *p* < 0.05 was considered statistically significant. In the analysis of clinical data, χ^2^ tests were used for comparing frequency data. GraphPad Prism 9 was used to generate graphs in the current study.

## 3 Results

### 3.1 Bioinformatics analysis of DEGs in GSE48558

#### 3.1.1 DEG screening

**Figure 2:**
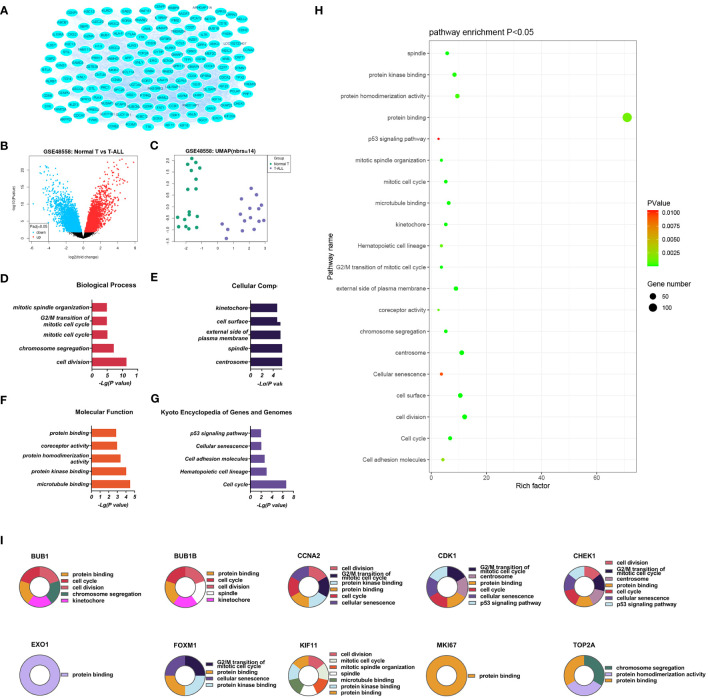
**(A)** Volcano plot of genes **(B)** DEGs between the control and T-ALL groups in the GSE48558 dataset. Symbols represent different genes, with red and green colors indicating upregulation and downregulation, respectively, using the criteria *p* < 0.05 and multiple change = 1. **(C)** Heatmap of DEGs. **(D-G)**. GO analysis, showing enrichment in the biological process **(D)**, cellular component **(E)**, and molecular function **(F)** categories, and KEGG pathway enrichment **(G)** of DEGs between the control and T-ALL/T-LBL groups. **(H)** Functions and pathways most likely to differ between the control and T-ALL/T-LBL groups. **(I)** Functions and regulatory pathways of the top 10 DEGs.

Based on the DEG screening criteria, a total of 208 DEGs were detected in the GSE48558 chip data (**Figure 2A**). Among them, 78 genes were upregulated, and 130 were downregulated (**Supplementary Tables 5, 6**). The identified DEGs between the control and T-ALL groups are shown in a volcano plot (**Figure 2B**), where red and green indicate significant upregulation and downregulation, respectively.

#### 3.1.2 Functional analysis of DEGs

Database for Annotation, Visualization and Integrated Discovery (DAVID) was used for GO analysis of the DEGs. The enrichment results are shown in **Figure 2**. Overall, 93 significant annotations were found in the biological process (BP) category, including those associated with cell division, chromosome aggregation, mitotic cell cycle, G2/M transition of the mitotic cell cycle, and mitotic spindle organization. Thirty-six annotations were seen in the cellular component (CC) category, including centrosome, spindle, external side of the plasma membrane, cell surface, and kinetochore. Simultaneously, 32 molecular functions (MF) were identified, most notably associated with microtubule binding, protein kinase binding, protein homeostasis activity, co-receptor activity, and protein binding. A total of 14 enriched pathways were identified by KEGG analysis, including the p53 signaling, cell adhesion molecules, hematopoietic cell lineage, cell cycle, and cellular sensitivity.

**Figure 3:**
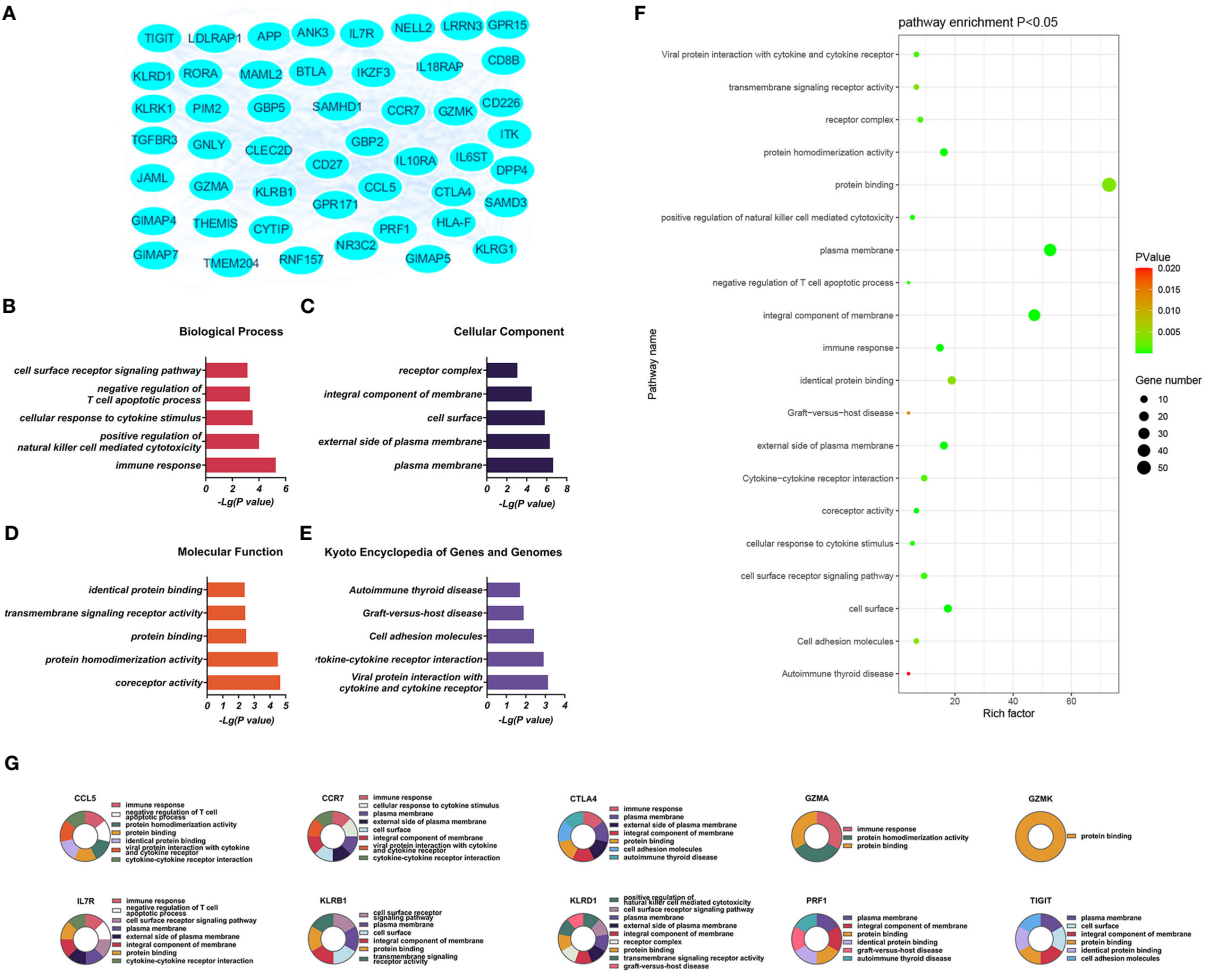
**(A)** Significantly upregulated DEGs between the control and T-ALL groups in the GSE48558 dataset. **(B–E)** Enrichment of upregulated DEGs in the GO, BP, CC, and MF categories, and KEGG pathway enrichment. **(F)** Functions and regulatory pathways show the most significant differences between the control and T-ALL/T-LBL groups. **(G)** Functions and regulatory pathways of the top 10 upregulated DEGs.

The significantly upregulated DEGs were enriched in 44 BPs, including positive regulation of natural killer (NK) cell-mediated cytotoxicity, immune response, cellular response to cytotoxic stimuli, negative regulation of T-cell apoptotic processes, and cell surface receptor signaling. Thirteen CCs were identified, including the external side of the plasma membrane, plasma membrane, integral component of membrane, cell surface, and receiver complex, and a further 13 MFs, including protein binding, co-receptor activity, transmembrane signaling receptor activity, protein homeostasis activity, and authentic protein binding were found. The most critical eight KEGG-enriched pathways were cell adhesion molecules, cytokine-cytokine receptor interaction, graft versus host disease, autoimmune thyroid disease, and viral protein interaction with cytosine-cytosine receptors (**Figure 3**).

**Figure 4:**
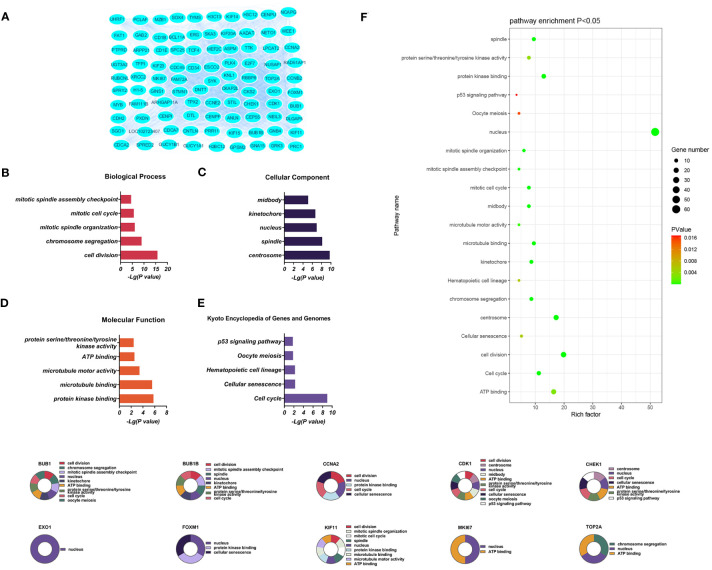
**(A)** Significantly downregulated DEGs between the control and T-ALL group from the GSE48558 dataset. **(B–E)** DEG enrichment in GO, BP, CC, MF categories, and KEGG pathways, respectively. **(F)** Functions and regulatory pathways show the most significant differences between the control and T-ALL/T-LBL groups. **(G)** Functions and regulatory pathways of the top 10 downregulated DEGs.

Downregulated DEGs in the GO BP category showed annotations mainly related to cell division, chromosome aggregation, mitotic spindle organization, mitotic cell cycle, and the mitotic spindle assembly checkpoint. Thirty-three CCs were identified, including centrosome, spindle, nucleus, kinetochore, and midbody. At the same time, enrichment in 24 MFs was found, mainly related to protein kinase binding, microtubule binding, microtubule motor activity, ATP binding, and protein serine/threonine/tyrosine kinase activity. KEGG analysis showed enrichment in 14 pathways, of which the cell cycle, cellular sensitivity, hematopoietic cell lineage, oocyte meiosis, and p53 signaling were the most significant (**Figure 4**).

The top five pathways showing the most significant enrichment were arranged according to their *p*-values and are listed in **Supplementary Tables 7–9**.

#### 3.1.3 PPI networks and network clustering modules

Three PPI networks were created using STRING and Cytoscape. The first was composed of overall DEGs, the second was composed of upregulated DEGs, and the third was composed of downregulated DEGs. The first PPI network of DEGs contained 150 nodes and 3116 paired interactions and showed a high topology score, which can thus be used as a significant network node. The top 10 genes are shown in **Supplementary Table 10**. The DEGs used for network construction were cyclin-dependent kinase 1 (*CDK1*), cyclin A2 (*CCNA2*), marker of proliferation Ki-67 (*MKI67*), topoisomerase II alpha (*TOP2A*), forkhead box protein M1 (*FOXM1*), exonuclease 1 (*EXO1*), kinesin family member 11 (*KIF11*), checkpoint kinase 1 (*CHEK1*), budding uninhibited by benzimidazoles 1 (*BUB1*), and *BUB1B*, all of which were overexpressed in T-ALL. The PPI network of the upregulated DEGs contained 49 nodes and 464 paired interactions. The topology score was high. The top 10 interactions are shown in **Supplementary Table 11**. The network was composed of 10 upregulated DEGs, namely, granzyme A (*GZMA*), interleukin 7 receptor (*IL7R*), *GZMK*, C-C chemokine ligand 5 (*CCL5*), C-C chemokine receptor 7 (*CCR7*), perforin (*PRF1*), T-cell immunoreceptor with Ig and ITIM domains (*TIGIT*), cytotoxic T-lymphocyte associated protein 4 (*CTLA4*), killer cell lectin-like receptor B1 (*KLRB1*), and *KLRD1*. The PPI network of downregulated DEGs included 94 nodes and 2498 paired interactions. **Supplementary Table 12** shows the top 10 genes. The DEGs in the network were *CCNA2*, *CDK1*, *KIF11*, *FOXM1*, *EXO1*, *TOP2A*, *MKI67*, *BUB1B*, *CHEK1*, and *BUB1*.

### 3.2 Models of markers in different groups determined by multi-parameter analysis

**Figure 5:**
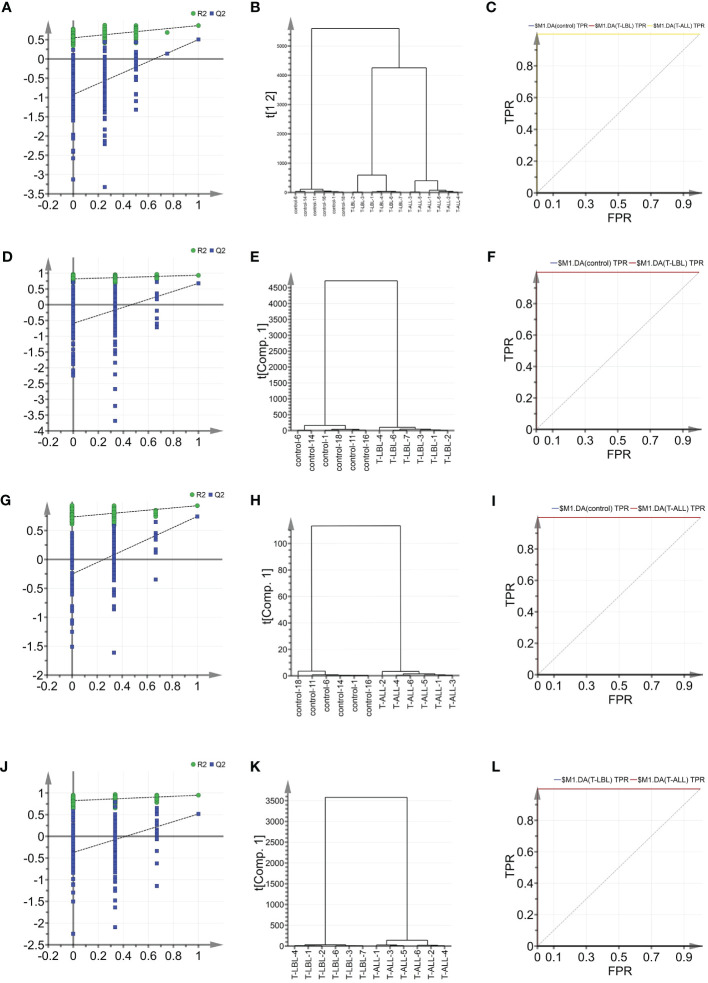
**(A)** OPLS-DA shows the permutation graph of the control, T-LBL, and T-ALL groups. **(B)** Cluster analysis of the three groups. **(C)** ROC curves of the three groups. AUC (control) = 1, AUC (T-LBL) = 1, AUC (T-ALL) = 1. **(D)** Permutation graph of control and T-LBL groups. **(E)** Cluster analysis of control and T-LBL groups. **(F)** ROC plot of control and T-LBL groups. AUC (control) = 1, AUC (T-LBL) = 1. **(G)** Permutation graph of control and T-ALL groups. **(H)** Cluster analysis of control and T-ALL groups. **(I)** ROC plot of control and T-ALL groups. AUC (control) = 1, AUC (T-ALL) = 1. **(J)** Permutation graph of T-LBL and T-ALL groups. **(K)** Cluster analysis graph of T-LBL and T-ALL groups. **(L)** ROC plot of T-LBL and T-ALL groups. AUC (T-LBL) = 1, AUC (T-ALL) = 1.

Indicators associated with the three groups were analysed using multiple parameter analyses to establish a method for characterising the control, T-LBL, and T-ALL groups. OPLS-DA was used with SIMCA-P software for detailed analysis and comparisons. The efficacy of the model based on the multi-parameter analysis was assessed using mutation (**Figures 5A, D, G, J**) and cluster analysis (**Figures 5B, E, H, K**) plots, and ROC curves (**Figures 5C, F, I, L**). The model’s validity was evaluated by permutation, which revealed a negative Q2 intercept on the Y-axis. This indicates that the model is valid and is not prone to overfitting (**Figures 5A, D, G, J**). Clustering analysis verified the discrimination between the different samples. ROC plots examined the effectiveness of the model by determining the area under the curve (AUC) values. AUC values close to 1 represent higher reliability of the identification method. The AUCs for the control vs T-LBL vs T-ALL model were AUC (control) = 1, AUC (T-LBL) = 1, and AUC (T-ALL) = 1 (**Figure 5C**). In the control vs T-LBL model, AUC (control) = 1 and AUC (T-LBL) = 1 (**Figure 5F**), in the control vs T-ALL model, AUC (control) = 1 and AUC (T-ALL) = 1 (**Figure 5I**) while in the T-LBL vs T-ALL model, AUC (control) = 1 and AUC (T-ALL) = 1 (**Figure 5I**), C (T-LBL) = 1, and AUC (T-ALL) = 1 (**Figure 5L**), indicating the accuracy of the results.

**Figure 6:**
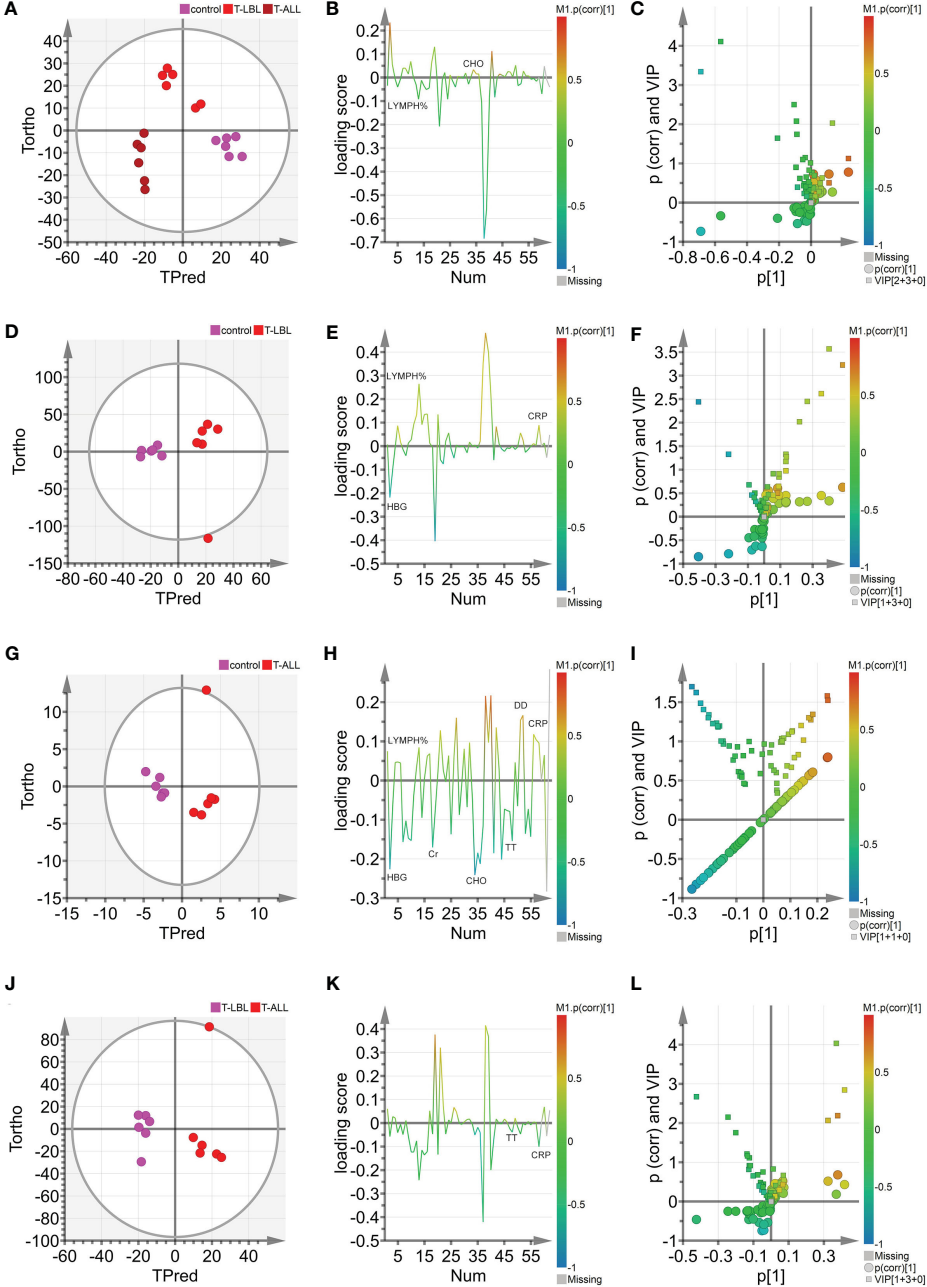
**(A)** Hotelling’s T2 ellipse score map of 95% confidence zone of control, T-LBL, and T-ALL groups identified by OPLS. **(B)** Loading plot of control, T-LBL, and T-ALL groups. **(C)** V + S plot of control, T-LBL, and T-ALL groups. **(D)** Hotelling’s T2 ellipse score map of 95% confidence zone of control and T-LBL groups. **(E)** Loading plot of control and T-LBL groups. **(F)** V + S plot of control and T-LBL groups. **(G)** Hotelling’s T2 ellipse score map of 95% confidence zone of control and T-ALL groups. **(H)** Loading plot of control and T-ALL groups. **(I)** V + S plot of control and T-ALL groups. **(J)** Hotelling’s T2 ellipse score map of 95% confidence zone of T-LBL and T-ALL groups. **(K)** Loading plot of T-LBL and T-ALL groups. **(L)** V + S plot of T-LBL and T-ALL groups.

OPLS-DA was used further to evaluate the associations between the different groups’ indicators and identify potential biomarkers. The scores are illustrated in **Figure 6A**, where the horizontal axis indicates the score values of the main components, and the vertical axis shows the scores of the orthogonal components. There is a clear separation and grouping of the samples, indicating differentiation between the three groups and demonstrating the method’s effectiveness in distinguishing between the indicators of the groups and providing a basis for further analysis. **Figures 6D, G, J** show the OPLS-DA scores for the control vs T-LBL, control vs T-ALL, and T-LBL vs T-ALL models, respectively. The locations of the samples on the horizontal axis and the clustering demonstrate the model’s effectiveness in differentiating between the samples according to their indicators. Both sets of samples in the figures are located in both the positive and negative areas of the X-axis. The distinction among the various sample sets is demonstrated in **Figures 6D, G, J**, illustrating the model’s efficacy in discerning the markers of the distinct samples. **Figure 6B** illustrates the loading plot that was used for the initial screening of the indicators for the different groups. The loading plot validates the findings shown in the score plot, illustrating the correlations between the indicators of the various samples. In the loading plot, the indicators located in the Y-axis’s positive region have higher values than those in the X-axis’s positive region. The negative halves of both axes of the loading plot also showed correspondence. Indicators such as LYMPH% and CHO significantly differed in the three groups, indicating that B12 levels in the controls were lower than those in the patient groups (**Figure 6B**). **Figure 6C** shows the OPLS-DA VIP plot, reflecting the association between the peak VIP values and correlation coefficients in the model. The red color in the peaks indicates higher correlation coefficients in the model. The V + S plot shows an integration of the VIP and correlation coefficient parameters characterizing the contribution of the indicators in the model. Individual points represent specific indicators with red color indicating higher correlation coefficients and thus greater contribution to the model. The blue color indicates a lower correlation and, thus, reduced contribution. Moreover, the greater distance between the indicator’s position and the center of the V + S plot indicates a more significant contribution to the model. To conduct a more thorough analysis of the factors that influence the model, V + S plots were utilized to identify potential biomarkers and examine the peaks for accurate distinction of the three groups (**Figure 6C**). The VIP values of distinct indicators in the classification model were assessed to determine the contribution of each indicator to the model. The primary indicator in the classification model was defined by its relevance. The V + S plot listed blood indicators in descending order of VIP value, with those with peaks of VIP > 1.0 and biological significance identified as potential biomarkers. OPLS-DA was conducted based on the control vs. T-LBL vs. T-ALL model by combining samples from the three groups in pairs (**Figures 6D–L**). **Figures 6D, G, J** represent the score plots of three pairwise combination models. The scatter points of the control, T-LBL, and T-ALL pairwise combinations are situated in the positive and negative regions, respectively, of the horizontal axis and show obvious separation. **Figures 6E, H, K** illustrate the loading plots of the three pairwise combinations. The control group exhibits lower levels of CRP and LYMPH, while displaying higher HGB values compared to the T-LBL group (**Figure 6E**). In addition, the controls had lower levels of CRP, DD, and LYMPH in comparison to the T-ALL patients, although their levels of Cr, HGB, CHO, and TT were higher (**Figure 6H**). Indicators such as TT and CRP help distinguish between T-LBL and T-ALL, as seen in **Figure 6K**. **Figures 6F, I, L**, respectively, represent the V + S plots of three pairwise combinations, providing an ordered list of blood indicators ranging from high to low VIP value that can be used for the identification of potential biomarkers in the models. A VIP value > 1.0, p (corr) > 0.35, and biological significance suggest a potential biomarker. A comprehensive consideration of the control vs. T-LBL vs. T-ALL model and three pairwise combination models using the parameters of VIP (VIP > 1.0), correlation coefficients, load, and distance from the center in the V + S graph identified various indicators affecting the sample classification, including CRP, LYMPH, DD, Cr, HGB, CHO, and TT. Indicators lacking significant differences were subsequently excluded from the list, resulting in the identification of EPO, FA, HGB, LDL, and TT as significant markers for distinguishing sample classification (**Supplementary Tables 3, 4**).

### 3.3 Serological data results of three groups

**Figure 7:**
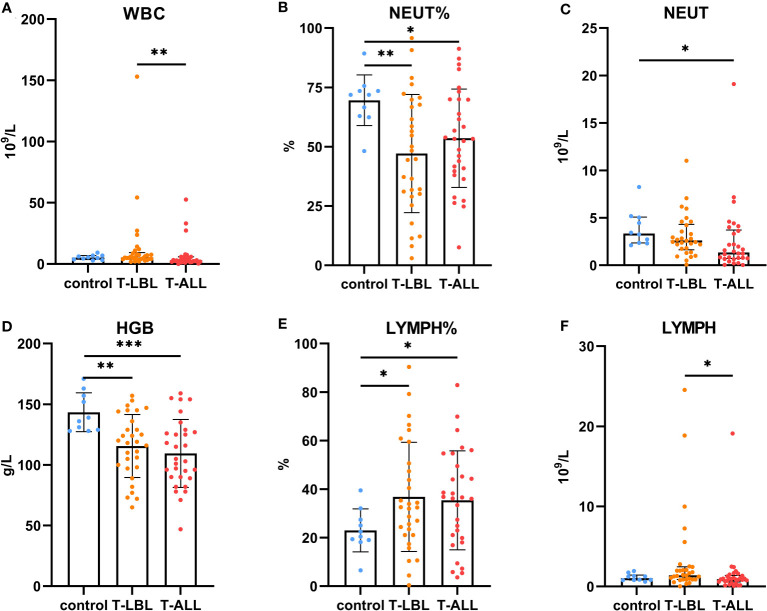
Comparison of routine blood measurements between the control, T-LBL, and T-ALL groups. Normally distributed data are shown as mean ± standard deviation, while non-normally distributed data are shown as median with quartile range. **(A)** WBC, **(B)** NEUT%, **(C)** NEUT, **(D)** HGB, **(E)** LYMPH%, **(F)** LYMPH. *p ≤ 0.05, **p ≤ 0.01, ***p = 0.001.

**Figure 8:**
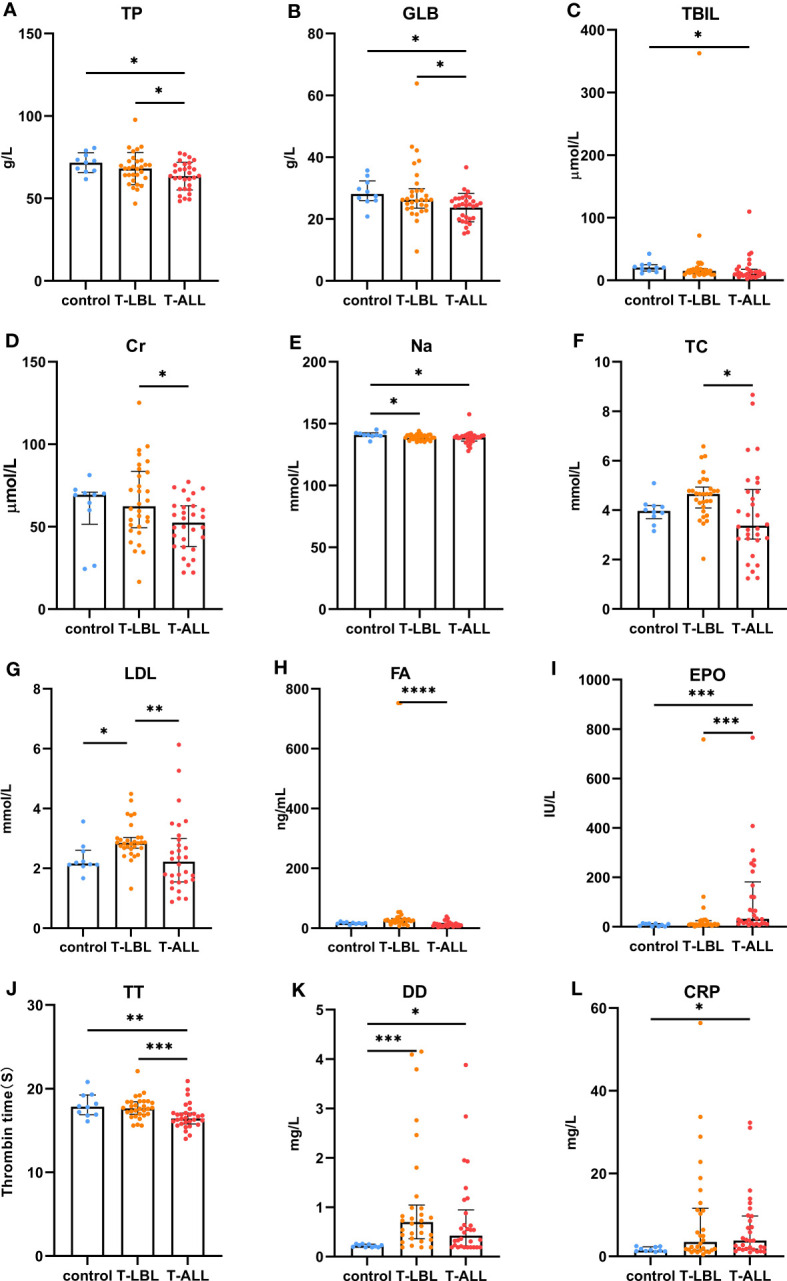
Comparison of peripheral blood biochemical indices between the control, T-LBL, and T-ALL groups. Normally distributed data are shown as mean ± standard deviation, while non-normally distributed data are shown as median with quartile ranges. **(A)** TP, **(B)** GLB, **(C)** TBIL, **(D)** Cr, **(E)** Na, **(F)** TC, **(G)** LDL, **(H)** FA, **(I)** EPO, **(J)** TT, **(K)** DD, **(L)** CRP. *P ≤ 0.05, **P ≤ 0.01, ***p = 0.001, ****p = 0.000.

Comparisons of the results of the peripheral blood routine analyses in the three groups (**Figure 7**) indicated significant differences in the white blood cell counts, neutrophil percentages and absolute values, hemoglobin levels, and lymphocyte percentages and absolute values (*p* < 0.05). In contrast, the comparison of biochemical indices (**Figure 8**) indicated marked differences in the levels of TP, GLB, TBIL, Cr, Na, CHO, LDL, FA, EPO, TT, DD, and CRP (*p* < 0.05). Comparison of clinical peripheral blood biochemical indicators analysis data among three groups showed no statistically significant differences in ASO, C3, C4, IgA, IgG, IgM, RF, F, Iron, ISAT, TIBC, UIBC, APTT, AT III, FDB, FIB, INR, PT, B12, Ca, CL, K, Mg, and P (*p* > 0.05), as shown in **Supplementary Figures S1, S2**.

Leukocyte counts in the peripheral blood were elevated in the T-LBL group relative to the T-ALL group (*p* = 0.004; **Figure 7A**). The neutrophil percentages in the T-LBL and T-ALL groups were reduced in comparison to controls (*p* = 0.006, *p* = 0.047; **Figure 7B**). The absolute value of neutrophils was lower in the T-ALL group than in the controls (*p* = 0.017; **Figure 7C**), while hemoglobin levels in both patient groups were significantly reduced (*p* = 0.004, *p* = 0.001; **Figure 7D**), as well as the percentage of peripheral blood lymphocytes (*p* = 0.026, *p* = 0.035; **Figure 7E**). Lymphocyte counts were elevated in the T-LBL group relative to the T-ALL group (*p* = 0.022; **Figure 7F**).

In terms of the biochemical indices, higher levels of ALB were observed in the control and T-LBL groups relative to the T-ALL group (*p* = 0.013, *p* = 0.047; **Figure 8A**), with a similar pattern seen for GLOB (*p* = 0.014, *p* = 0.039; **Figure 8B**). TB levels were higher in the controls relative to the T-ALL group (*p* = 0.020; **Figure 8C**), while CR levels were elevated in the T-LBL group relative to T-ALL (*p* = 0.048; **Figure 8D**). Furthermore, Na levels were reduced in both the T-LBL and T-ALL groups (*p* = 0.036, *p* = 0.021; **Figure 8E**), while CHO concentrations were higher in the T-LBL group compared to T-ALL (*p* = 0.013; **Figure 8F**), LDL concentrations in the T-LBL group were increased (*p* = 0.032, *p* = 0.005; **Figure 8G**), and FA was raised in T-LBL rather than T-ALL (*p* = 0.000; **Figure 8H**). The EPO concentrations were significantly elevated in T-ALL patients but not in others (*p* = 0.001, *p* = 0.001; **Figure 8I**). The TT levels were decreased in T-ALL patients (*p* = 0.008, *p* = 0.001; **Figure 8J**), DD concentrations in both the T-LBL and T-ALL groups (*p* = 0.001, *p* = 0.048; **Figure 8K**), and CRP was elevated in T-ALL in comparison to controls (*p* = 0.044; **Figure 8L**).

## 4 Discussion

This study demonstrated that T-ALL is more likely to have relatively immature immunological phenotypes compared to T-LBL, but there is some similarity between the two. Interestingly, T-ALL and T-LBL are part of the same biological disorder, and their categorization is entirely arbitrary. When the patient only presents a tumor mass without bone marrow and blood involvement, it is diagnosed as lymphoma. However, if there is a tumor mass along with a smaller percentage (≤ 25%) of bone marrow infiltration, it is still considered lymphoma rather than leukemia. On the other hand, if there is extensive bone marrow involvement (≥ 25%) and blood involvement, it is diagnosed as lymphoblastic leukemia. Here, the white blood cell, red blood cell, lymphocyte counts, and absolute and percentage neutrophils did not differ between the T-ALL and T-LBL groups. However, more significant reductions were seen in T-ALL patients than controls, indicative of increased white blood cell counts typical of T-ALL. Even with the same white blood cell counts and tumor loads, T-ALL patients show reduced hematopoiesis compared to ALL types. In contrast to the controls, T-ALL patients had lower levels of ALB, GLOB, CR, CHO, LDL, and erythropoietin in the peripheral blood, while T-LBL patients had higher CHO, LDL, and folate levels. Based on the results of the multi-parameter analysis, HGB, EPO, TT, and DD were considered essential indicators for sample classification, as depicted by **Figure 1**; **Supplementary Tables 1, 2**.

EPO is a glycoprotein hormone that can increase the production of red blood cells. Tissue oxygenation governs the synthesis of EPO, which is responsible for the prompt generation of a significant quantity of red blood cells to alleviate the symptoms of hypoxia (6). It was observed that the T-ALL patients had more severe anemia than those with T-LBL. In hypoxic environments, the levels of EPO gradually rise due to the activity of the transcription factor complex HIF. This enables the body to adjust progressively to hypoxic situations like anemia, and it aligns with the finding that elevated levels of EPO are found in patients with T-ALL. It was speculated that supplementation of EPO to treat anemia in T-ALL patients requires assessing the risks and benefits. The degradation of cross-linked fibrin produces DD, and increased concentrations can result in both embolism and coagulation in the vasculature; thus, the determination of DD levels is useful in evaluating thrombotic disease. In the present study, significantly higher levels of DD were seen in both T-LBL and T-ALL patients (*p* = 0.001, *p* = 0.048). The TT is one of the screening indicators for coagulation, anticoagulation, and fibrinolytic functions. TT is a measure of the ability of exogenous thrombin to hydrolyze FIB, and can be used to evaluate the function of FIB and determine whether there is a defect in its function. Although the peripheral blood FIB levels did not show any significant changes in the present study, it was found that the TT time in T-ALL patients was considerably shorter than that in the controls and T-LBL patients (*p* = 0.008, *p* = 0.001). Thus, there was speculation that people with T-ALL are more prone to developing coagulation abnormalities compared to patients with T-LBL. However, there were no statistically significant differences in peripheral blood F, Iron, and ISAT levels among the three groups in this study. Ferroptosis has garnered interest as a potential broad-spectrum anti-cancer approach in leukemia studies, as it is a kind of cell death that relies on iron regulation. Studies have shown that adult T-cell leukemia/lymphoma (ATL) is a highly invasive malignant tumor caused by human T-cell leukemia virus type I (HTLV-1). Wang et al. used bioinformatics analysis and dataset GSE33615 to identify 46 DEGs associated with ferroptosis and 26 autophagy associated DEGs in ATL cells. These DEGs were associated with various cellular responses, chemical stress, and iron related pathways. Autophagy-related DEGs were associated with autophagy, cell apoptosis, nucleotide oligomerization domain (NOD)-like receptor signaling, tumor necrosis factor (TNF) signaling, and insulin resistance pathways. PPI network analysis revealed 10 central genes and related biomolecules. Researchers found that the ATL specific ferroptosis signal is unique by comparing the ferroptosis characteristics of other T-cell lymphomas. This study establishes an innovative link between ATL treatment and ferroptosis, providing a promising pathway for novel treatment strategies for ATL (7).

T-LBL and T-ALL are relatively rare and highly aggressive hematological malignancies. Advances in research have led to the development of many therapeutic drugs, including hypomethylation drugs, drug-antibody conjugates, kinase inhibitors, and allogeneic hematopoietic stem cell transplantation (HSCT) (8, 9). However, over 50% of patients fall below the criteria for intensive chemotherapy. In addition, induced remission chemotherapy and HSCT have also led to a large number of treatment-related complications, recurrence, and even death. These represent significant challenges for diagnosing, managing, and prognostic predicting hematological malignancies, requiring intensive further investigation. In clinical practice, the NHL standard treatment plan CHOP can be chosen for the initial treatment of T-LBL without bone marrow or other organ invasion. However, once a lump or involvement of bone marrow and other organs occurs during the treatment period, treatment for T-ALL must be instigated. Tumor heterogeneity may be one of the reasons for the failure of treatment for refractory T-ALL. Targeted and total RNA sequencing methods are effective techniques for identifying gene fusion events caused by chromosomal rearrangements. These methods can detect various types of fusions, including those defined by the World Health Organization and the International Consensus Classification of Hemolymph Tumors, as well as cryptic fusions that are often missed by traditional cytogenetics. Additionally, these methods can identify rare cytogenetic fusion events that may be responsible for the lack of response to targeted therapy. The samples of targeted RNA NGS studied by Tsai, H.K., et al. were selected from total nucleic acids extracted from bone marrow, peripheral blood, extramedullary disease sites, or cell lines and subjected to bioinformatics processing. Outlier analysis revealed a rare deletion of the *NOTCH1* gene in T-ALL, and screening of *NOTCH1* subtypes revealed two cases of T-ALL with abnormal expression of e2e28 (10). Tan, K. et al. identified a subset of bone marrow progenitor-like (BMP-like) leukemia associated with treatment failure and poor overall survival through a comprehensive analysis of T-ALL mother cells and normal T cell precursors. Using a large amount of RNA sequencing data from over 1300 patients, single-cell derived molecular features of BMP-like mother cells were used to predict adverse outcomes for multiple T-ALL subtypes in two independent patient cohorts. This study defined the mutation status of BMP-like T-ALL and found that the *NOTCH1* mutation additionally drives T-ALL mother cells away from the BMP-like state. The data suggests that the *NOTCH1* mutation status is a key biomarker of traditional treatment response (11). The results of this work can serve as a clinical guide for studying efficient therapy alternatives for T-ALL/T-LBL and, therefore, enhancing unfavorable prognosis (12, 13).

T-LBL and T-ALL arise from the lymphoid tissue in the thymus, originating from the same tumor clone and representing the same biological disease. Therefore, their categorization into separate entities is entirely arbitrary. Both conditions may develop due to anomalies in antigen receptor genes, chromosomal abnormalities, inactivation of tumor suppressor genes, and activation of oncogenes. This study aimed to identify abnormally expressed genes at the molecular level. The data on T-cell hematological malignancies from separate research in the GEO database were collected and subjected to normalization preprocessing. A total of 208 genes showing significant differential expression were identified between the controls and T-ALL patients; 78 of these DEGs were upregulated, with 130 downregulated. The DEGs were found to be linked to processes involving cell division, chromosome aggregation, mitotic cell cycle, microtubule binding, protein kinase binding, cell adhesion molecules, cellular senescence, and p53 signaling. Bioinformatics analysis, including PPI networks and identification of key genes, identified 10 key DEGs that were expressed at significantly higher levels in T-ALL/T-LBL patients, namely, *CDK1*, *CCNA2*, *MKI67*, *TOP2A*, *FOXM1*, *EXO1*, *KIF11*, *CHEK1*, *BUB1B*, and *BUB1*. A further 10 key DEGs showing significant downregulation in T-ALL/T-LBL patients were also identified, namely, *GZMA*, *IL7R*, *GZMK*, *CCL5*, *CCR7*, *PRF1*, *TIGIT*, *CTLA4*, *KLRB1*, and *KLRD1*. The top 10 key DEGs identified from the network analysis and multi-parameter and statistical analyses explore potential biomarkers or key DEGs that could be used in clinical applications.

Ten DEGs that exhibited increased expression in T-ALL were found. Extensive evidence supports the notion that the aberrant expression of genes involved in tyrosine phosphorylation pathways is strongly connected with several human cancers. Cyclin-dependent kinases (CDKs) are a kind of kinase that specifically phosphorylates serine and threonine residues. They are highly regarded as potential targets for cancer therapy (14). CDK1 is a crucial CDK for cell cycle progression, associated with mitotic progression and cell division, such as cytoskeletal recombination, nuclear membrane rupture, chromosome condensation, mitotic spindle assembly, chromosome separation, and cytoplasmic division (15). Studies have shown that mutations in certain genes can induce overexpression of CDK1, promote tumor cell growth, migration, or invasion, and promote the occurrence of leukemia (14). CDK1 was also shown to be over-expressed in T-ALL/T-LBL. Therefore, CDK1 is a potential therapeutic target for cancer treatment (16). This gene is associated with the initiation of mitosis, which may exacerbate disease progression by promoting the cell cycle and inhibiting the P53 pathway (17, 18). *CCNA2* encodes a strongly conserved cell cycle-associated protein responsible for cell cycle regulation (19, 20). *CCNA2* belongs to the highly conserved cyclin family, located on human chromosome 4 and expressed in almost all human body tissues. Generally speaking, the protein encoded by *CCNA2* can activate CDK2, which may participate in the occurrence and progression of various tumors by affecting epithelial-mesenchymal transition (EMT), metastasis, and may enhance cancer invasion, recurrence, and chemotherapy resistance (21). MKI67 plays a role in controlling the separation of chromosomes during cell division and is a reliable marker for identifying the growth of cancer cells. It may be utilized to evaluate the malignancy and prognosis of the disease (22, 23). MKI67 is a nuclear protein expressed in the proliferating cell nucleus, closely related to cell proliferation, and plays an important role in the formation of mitotic spindles and mitosis. Its abnormal expression is closely related to the onset and development of tumors (24). The expression of MKI67 represents the state of cell proliferation and plays an important role in tumor migration, invasion, and progression (25). Studies have shown that P53 exerts inhibitory effects on the Ki-67 promoter by regulating the P53 and sp1 dependent pathways, and have also demonstrated the close relationship between Ki-67/MKI67 and the P53 signaling pathway (26). TOP2A is involved in chromosome aggregation, the separation of chromatids, and reducing torsional stress during DNA replication and transcription (27, 28). TOP2A is an important nuclear protein that is essential for cell division and highly expressed during mitosis, as it is responsible for chromosome aggregation and separation. It can regulate and modify the topological state of DNA during transcription, promote chromatid separation, chromosome condensation, reduce torsional stress during transcription and DNA replication, alter the structure of DNA, and is related to cell invasion, migration, and cell cycle (29). TOP2A, as a target of chemotherapy drugs, has been proven to be widely involved in the invasion and prognosis of various human cancers (30). FOXM1 is a Forkhead box transcription factor that participates in various cellular processes, such as proliferation, cell cycle progression, cell differentiation, DNA damage repair, tissue homeostasis, angiogenesis, apoptosis, and redox signal transduction (31). FOXM1 is a promising candidate for therapeutic intervention in several human malignancies (32) and has a role in developing and treating several immune-related and vascular disorders (33). FOXM1 encodes a protein associated with the mitotic cell cycle (34, 35). The product of EXO1 gene has exonuclease activity, and its function is mainly involved in mismatch repair and recombination. It is upregulated in tumor tissues, including gastric cancer, lung adenocarcinoma, and ovarian cancer (36–38). Tumor occurrence and development can be mediated through involvement in hypoxia related pathways, which are usually caused by an imbalance in the supply and demand of nutrients in the tumor microenvironment. Hypoxia is a common physiological hallmark of most tumors, especially those with malignant traits like metastasis and invasion. However, the prevalence and severity of hypoxia might differ among different patient groups (39–41). Under hypoxic conditions, cancer cells secrete angiogenic factors to promote abnormal angiogenesis. In addition, hypoxia increases the malignancy of tumors and allows cancer cells to invade and metastasize, leading to the insensitivity of cancer cells to chemotherapy or radiotherapy (37, 42). In contrast, the depletion of EXO1 inhibits cell proliferation, migration, and invasive activity of tumors (37). This study determined that the concentrations of HGB and EPO in the peripheral blood of patients with T-ALL/T-LBL are clinically significant in assessing these conditions. It has been proposed that T-ALL/T-LBL patients may experience hypoxia in the tumor microenvironment due to aberrant expression of the *EXO1* gene. The functions of *KIF11* gene products include chromosome localization, centrosome separation, and the establishment of bipolar spindles during mitosis (43, 44). *KIF11* has a low expression level in most tissues, but a high expression level in immune related tissues such as thymus, bone marrow, tonsils, and lymph nodes. On the contrary, *KIF11* is usually expressed at high levels in cancer cell lines and promotes tumor occurrence and progression (45). In addition, the function of *KIF11* in the lymphatic system suggests the possibility that *KIF11* may indirectly affect tumor progression *via* immune functions. Cytological experiments were conducted to determine the regulatory effects of KIF11 on the proliferation and cell cycle progression of ALL *in vitro* (46). In addition, the KIF11 interaction and co expression network are mainly involved in the regulation of cell cycle, cell division, p53 signaling pathway, DNA repair and recombination, chromatin tissue, antigen processing and presentation, and drug resistance. In addition, tumor related neutrophils can support tumor progression by stimulating tumor cell invasion, migration, and movement, promoting angiogenesis, and regulating other immune cells (47–50). KIF11 may play multiple roles in tumors by influencing the infiltration of neutrophils. The protein encoded by *CHEK1* gene affects DNA repair and mitosis. Many solid tumors, especially those lacking TP53, significantly rely on CHEK1 mediated cell cycle arrest (51), and CHEK1 expression levels are upregulated in various cancers (52). The *BUB1B* and *BUB1* genes encode mitotic checkpoint proteins involved in chromosomal segregation (53, 54). BUB1 has been demonstrated to be a pivotal protein in the process of mitosis, playing a role in the initiation and progression of several types of malignancies (55–57). BUB1 vs. TGF-β dependent signaling has important effects (57), which can block the phosphatidylinositol 3-kinase (PI3K)/AKT and extracellular signal-regulated kinase (ERK) signaling pathways, significantly inhibit cell proliferation, tumor growth, cell migration, and invasion (58). The bioinformatics analyses showed that p53 is closely involved in the division cycle in T-ALL/T-LBL cells. Cellular levels of p53 are normally low, as it is a tumor suppressor. In cancer cells, however, expression is significantly increased, often accompanied by mutational burden. Patients with high p53 expression usually respond poorly to treatment. Therefore, the high expression of CDK1 and CHEK1 in T-ALL patients may be related to their poor prognosis. In summary, abnormalities in any stage of the mitotic cell cycle can lead to chromosomal instability, including increased expression of the *CDK1*, *CCNA2*, *MKI67*, *TOP2A*, *FOXM1*, *KIF11*, *CHEK1*, *BUB1B*, and *BUB1* genes in T-ALL/T-LBL patients, might be linked with the unfavorable prognosis.

The present work identified 10 key downregulated DEGs in T-ALL. GZMA participates in typical apoptotic features such as chromatin concentration, phosphatidylserine inversion, nuclear fragmentation, and reduced mitochondrial transmembrane potential in cells (59, 60). IL7R encodes the interleukin-7 (IL-7) receptor. IL-7 serves as a growth factor in the process of hematopoiesis, promoting the proliferation and differentiation of hematological malignancies and stimulating immunological responses in fully developed T-cells. The signal transduction of IL-7R is essential for the input, proliferation, and survival of early T-cell progenitor cells. IL7R is activated by the ligand IL7, and over 10% of T-ALL cases have acquired mutations in IL7R, leading to continuous activation of Janus kinase/signal transducers and activators of transcription (JAK/STAT) signaling cascade (61). The downregulation of IL7R in T-ALL/T-LBL might cause a rise in tyrosine kinase activity inside the leukemia cells, leading to continuous stimulation of cytokine receptor signaling pathways and ultimately culminating in significant impairments in T-cell function (61, 62). The *GZMK* gene product is a serine protease produced in cytoplasmic granules in cytotoxic lymphocytes (63, 64). The *PRF1* gene encodes perforin 1, involved in the formation of pores in membranes and T- and NK-cell mediated cell lysis of various cell types. Perforin is a pore-forming protein stored in acidic secretory granules of cytotoxic lymphocytes. Perforin is crucial for immune homeostasis and tumor immune surveillance. The mediated cytotoxicity is essential for eliminating altered cells and cells harboring intracellular infections. Additionally, it suppresses immunological responses by triggering apoptosis of effector lymphocytes and antigen-presenting cells. Perforins have also been shown to play an important role in NK cell-mediated inhibition of tumor metastasis and control of carcinogen-induced sarcoma growth. Jaworowska et al. investigated the impact of perforin gene mutations on clinical outcomes in children with ALL. This study suggests that mutations in the perforin gene alter the mortality rate of childhood ALL (65). In T-ALL/T-LBL, reduced expression of the cytotoxic *GZMK* and *PRF1* genes adversely affects killing by cytotoxic T-lymphocytes as well as the immune response (65, 66). Although the lymphocyte percentages were higher in T-ALL/T-LBL patients compared to controls, the absolute lymphocyte level did not change (as shown in **Figures 7E, F**). Granulase-mediated cell death is a key mechanism by which cytotoxic lymphocytes eliminate malignant cells in anti-tumor immunity (67). GZMK (formerly known as trypsin-2) is involved in one of the mechanisms by which cytotoxic T-lymphocytes and NK cells induce tumor cell apoptosis. GZMK and GZMA show significant homology, being situated in the same chromosomal locus, displaying a high degree of conservation in their sequence, and sharing the same cleavage specificity resembling trypsin activity. Research has demonstrated that GZMK is markedly upregulated in patients who do not have recurrence. Furthermore, the level of GZMK expression might serve as a valuable indicator for identifying individuals with a favorable prognosis in high-risk recurrence groups (64). It was speculated that reduced expression of GZMK in the patients was associated with decreased cytotoxic T-lymphocyte activities. The *CCL5* and *CCR7* genes are involved in the expression of chemokines and their receptors and in immune regulation and inflammatory processes. A link exists between the formation of tumors and the movement of cells in response to chemical signals (chemotaxis) in the body. In this study, individuals with T-ALL had elevated levels of CRP compared to the control group (*p* = 0.044). It was proposed that T-ALL patients may be more prone to inflammatory mediator dysfunction. The *TIGIT* gene encodes T-cell immunoglobulin and immune receptor tyrosine inhibitory motif domain (TIGIT) receptors, which can promote tumor cell immune evasion (68, 69). The chimeric antigen receptor (CAR) T-cell therapy using brexucabtagene autotoll (BA) can induce remission in many patients with mantle cell lymphoma (MCL). BA is the only FDA-approved CAR T-cell therapy for MCL. Following the recurrence of BA, there is a drop in the number of T-cells, particularly cytotoxic T-lymphocytes (CTL), in non-tumor cells, while the proportion of myeloid cells increases accordingly. Following recurrence, there was a notable increase in TIGIT expression on depleted T-cells. Additionally, CTL tumor cells also displayed TIGIT expression after recurrence, resulting in a heightened interaction between TIGIT on tumor cells and CD155/PVR on monocytes. The expression of TIGIT on tumor cells is particular to MCL, and when paired with targeted TIGIT, it can limit the recurrence of CAR T-cells. This ultimately promotes long-term progression-free survival in MCL patients (68). *CTLA4* belongs to the immunoglobulin superfamily and encodes a protein that inhibits T-cell activity, thus providing negative regulation (70, 71). In the tumor microenvironment of patients with T-ALL/T-LBL, the immunosuppressive components *TIGIT* and *CTLA4* show reduced expression. Single-cell RNA sequencing was performed on T-cells isolated from the peripheral blood of healthy individuals and patients with B-cell acute lymphoblastic leukemia (B-ALL). Two depleted T-cell populations were specifically identified in B-ALL patients, characterized by upregulation of *TIGIT*, *PDCD1*, *HLADRA*, *LAG3*, and *CTLA4* (Wang and Chen et al., 2021). Therefore, it was speculated that these patients will likely respond poorly to immune checkpoint drugs. *KLRB1* is a gene that codes for a killer lectin-like receptor (KLR) receptor. This receptor belongs to the superfamily of C-type lectins and is associated with good prognosis. It encodes *CD161* and is present on T-cells and specific subsets of NK cells (72, 73). KLRD1 (CD94) is expressed on NK cells, which appears to regulate NK cell functions (74, 75). Cancer immunotherapy surpasses traditional therapies in terms of its ability to selectively target cancer cells while minimizing adverse effects on healthy cells. T-cells have traditionally been the primary focus, but NK cells possess comparable capabilities. The T-ALL NK cells have a deficiency in the two prominent genes, *CD69* and *KLRD1*, and the consequences of their deletion on T-ALL remain uncertain. The ALL subtype can hinder the activity of NK cells through numerous mechanisms (74). It was speculated that KLRB1 and KLRD1 show reduced expression in the tumor microenvironment of patients with T-ALL/T-LBL, which can lead to immune response deficits.

To summarize, the tumor microenvironment of T-ALL/T-LBL exhibits impaired inflammatory mediators and functional abnormalities in T-cells, NK cells, and immunological checkpoints. The expression levels of *GZMA*, *IL7R*, *GZMK*, *CCL5*, *CCR7*, *PRF1*, *TIGIT*, *CTLA4*, *KLRB1*, and *KLRD1* are decreased in these individuals, which is associated with an unfavorable prognosis and inadequate long-term clinical immunological response.

Although scholars have an increasing understanding of the role of the aforementioned key genes in tumor-related diseases, many areas still need to be explored in terms of their activation, regulation, benefits, and improved cancer immunotherapy or reduced treatment-related toxicity. Moreover, the mechanisms and specific applications of these genes in T-ALL/T-LBL patients still require further in-depth research. Targeted therapy shows potential as an effective treatment for Retired/Recovery (R/R) T-ALL. According to a recent study by ASH 2023, efforts have been made to customize R/RT ALL therapy by focusing on specific mutated genes and cellular signaling pathways that are excessively active in cancer cells. These endeavors have resulted in some positive outcomes. Researchers have initiated the ALL-TARGET project as a specialized medical platform for R/R T-ALL and T-cell lymphoblastic lymphoma (T-LL). Its primary purpose is to assess therapy choices by considering gene mutation lineages and alterations in intracellular signaling networks (76). T-ALL/T-LBL shows high heterogeneity, and despite some advances in the treatment of hematological tumors, patient prognosis remains poor. Current research on T-ALL/T-LBL has focused mainly on identifying the underlying pathways, indicating anti-leukemia therapy’s future (77–79). Targeted therapy for T-ALL/T-LBL could involve multiple aspects, including targeting apoptosis, RTKs, hedgehog-associated signaling, mitochondrial metabolism and respiration, transcriptional control, and immunotherapy (80–83). In addition, leukemia stem cells (LSCs) are also involved in the persistence and development of diseases. The current investigation employed serum metabolomics analysis and statistical techniques to examine these indicators. Additionally, bioinformatics analysis tools were utilized to identify crucial genes, compare variations in biomarkers across samples, and determine appropriate peripheral blood biomarkers for clinical utilization. Additionally, this serves as a foundational framework for future research endeavors.

This study re-examined the dataset, especially for errors, outliers, or duplicate samples. The conclusion was that all raw data was accurate and performed well on the training set. Furthermore, cross-validation methods were used to divide the dataset into multiple non-overlapping subsets for various training and validation. In summary, it was determined that the model in this study did not show overfitting by observing the performance of the training and validation sets, comparing training and validation errors, using cross-validation, and observing the learning curve. The samples for this study were from a specialized hospital for hematology, and all samples were collected with high reliability and authenticity. This study aims to recognize the increasing importance of molecular genetic analysis in the diagnosis and prognosis of T-ALL/T-LBL. Hence, in the investigation of lymphoblastic lymphoma, the diagnosis of T-ALL and T-LBL was established by employing conventional histology, immunophenotype, conventional cytogenetic analysis, fluorescence *in situ* hybridization, and other techniques and meticulously categorizing them.

After introducing intensive therapy, the cure rates of T-ALL/T-LBL patients in children and adults reached over 60% and 80%, respectively. However, the prognosis of patients with primary drug resistance and refractory recurrence is still poor. The current research objective was to understand the pathogenesis of T-ALL/T-LBL, search for new therapeutic targets, help develop drugs with lower toxicity, more potent efficacy, more specific effects with improved cure rate, and reduce pain and complications. The research design of this study was based on bioinformatics analysis, combined with patient serological analysis data for validation, which increases the reliability of the results. Despite the limitation of sample size, the study validated the results with multiple parameter analysis and statistical methods. It combined the screened genes with the hematological and biochemical indicators obtained from statistical analysis to explore potential therapeutic targets for T-ALL/T-LBL. This study is only an initial investigation; further support regarding sample size and other data is required. However, it is essential to note that the samples used in this study were obtained from a specialized hospital that focuses on hematology. These samples were carefully selected and diagnosed, ensuring the reliability of the results, which align with the research goals. However, more extensive investigations supporting *in vitro* cell functional tests are needed to confirm the validity of these findings.

Limitations of this study: Patient histories of past symptoms, medication, smoking, and alcohol consumption were not addressed, which may have influenced the experimental results. Future investigations should include increased sample sizes and standardized data collection methods. The typical manifestation of T-ALL is increased white blood cell counts. However, in the present study, these counts did not differ significantly between T-ALL and T-LBL, nor did the blood biochemical indices (**Supplementary Figures 1–3**).

## 5 Conclusions

Despite new treatments for T-ALL/T-LBL, including small-molecule drugs and targeted treatments, the prognosis remains poor. Combining serum metabolomics multi-parameter analysis and statistical results, it was found that the peripheral blood levels of HGB, EPO, TT, DD, and CRP may have clinical value for evaluating the disease. Gene enrichment analyses identified key genes, including *CDK1*, *CCNA2*, *MKI67*, *TOP2A*, *FOXM1*, *EXO1*, *KIF11*, *CHEK1*, *BUB1B*, *BUB1*, *GZMA*, *IL7R*, *GZMK*, *CCL5*, *CCR7*, *PRF1*, *TIGIT*, *CTLA4*, *KLRB1*, and *KLRD1* which may modulate T-ALL pathogenesis. These genes were associated with abnormal mitotic cell cycles, chromosomal instability, dysfunction of inflammatory mediators, functional defects in T-cells, NK cells, and immune checkpoints. They were linked to poor prognosis and treatment response. While more extensive experimental research is required, this work can serve as a clinical point of reference for investigating efficient therapy choices for T-ALL/T-LBL and the precise targeting of particular cell types to enhance patient prognosis. Furthermore, an additional comprehensive study is needed to investigate the processes and particular uses of these genes in patients with T-ALL/T-LBL. The future efforts will be directed towards identifying and characterizing recurring anomalies in specific leukemia pathways, as well as oncogenes and tumor suppressor genes that may be used as diagnostic biomarkers for T-ALL. In addition, the focus is on exploring the practical use of the intricate genomic map of T-ALL to develop and evaluate novel and tailored therapeutic approaches.

## Data availability statement

The datasets presented in this study can be found in online repositories. The names of the repository/repositories and accession number(s) can be found in the article/**Supplementary Material**.

## Ethics statement

The studies involving humans were approved by The Ethics Committee of Hematology Hospital of the Chinese Academy of Medical Sciences (Institute of Hematology, Chinese Academy of Medical Sciences). The studies were conducted in accordance with the local legislation and institutional requirements. Written informed consent for participation in this study was provided by the participants’ legal guardians/next of kin.

## Author contributions

YR: Conceptualization, Data curation, Formal analysis, Writing – original draft. HL: Conceptualization, Data curation, Writing – original draft. YH: Conceptualization, Writing – original draft. YM: Methodology, Writing – review & editing. RL: Methodology, Writing – review & editing. JuQ: Methodology, Writing – review & editing. LW: Methodology, Writing – review & editing. JiQ: Methodology, Writing – review & editing. YL: Methodology, Writing – review & editing. YX: Resources, Writing – review & editing. LH: Resources, Writing – review & editing. SW: Resources, Writing – review & editing. XK: Methodology, Writing – review & editing. YZ: Supervision, Writing – review & editing. QZ: Supervision, Writing – review & editing. GZ: Funding acquisition, Project administration, Supervision, Writing – review & editing.
