# Supplementary material for: Key candidate genes and pathways in T lymphoblastic leukemia/lymphoma identified by bioinformatics and serological analyses
Source: Front Immunol. 2024 Feb 23;15:1341255. doi: 10.3389/fimmu.2024.1341255 (PMC10920334; doi:10.3389/fimmu.2024.1341255)
Supplement: Supplementary file 1 [file DataSheet_1.docx]

Supplementary Material


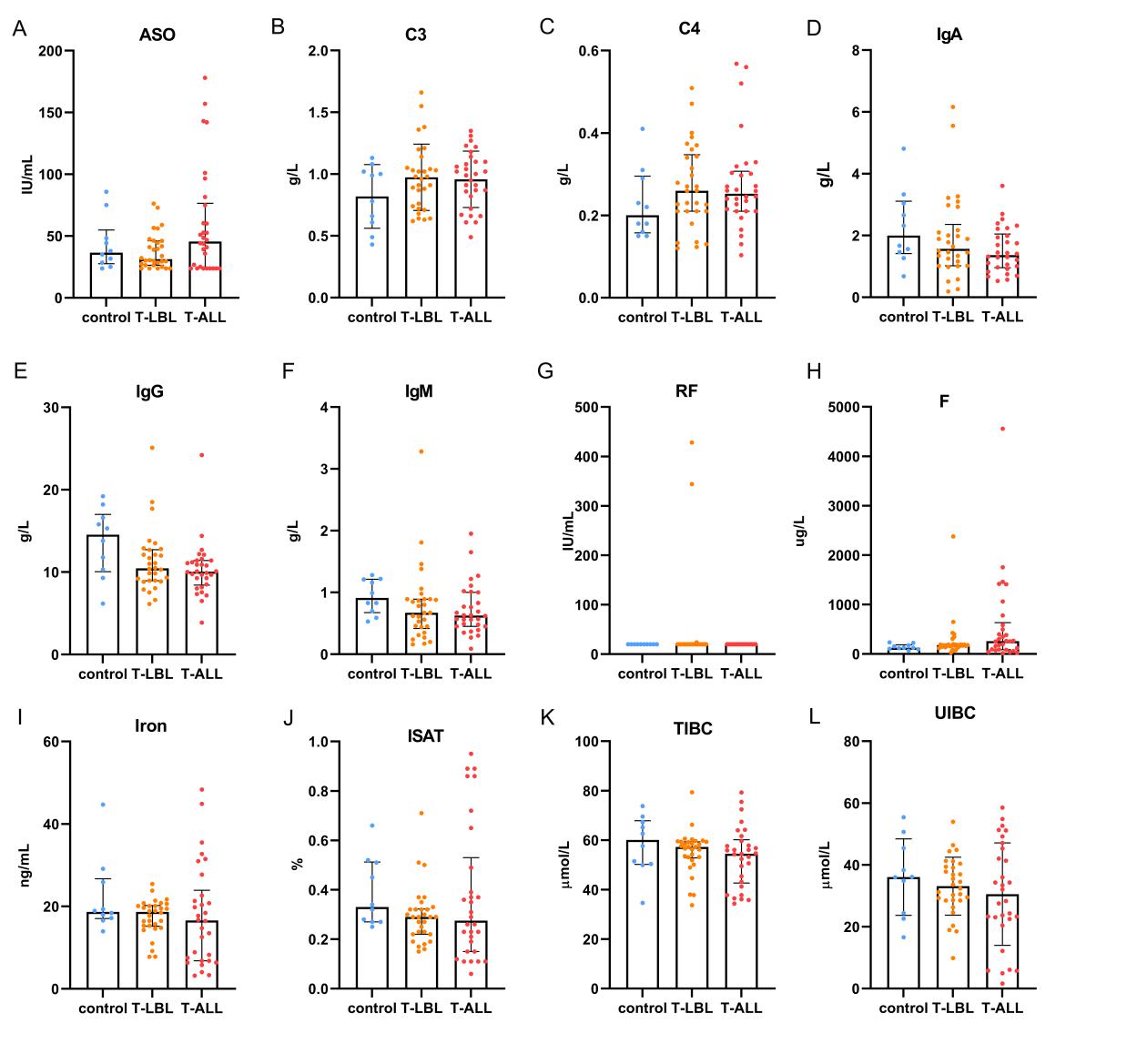


**Supplementary** **Figure 1.** Comparison of peripheral blood immune and iron indicators between the control, T-LBL, and T-ALL groups


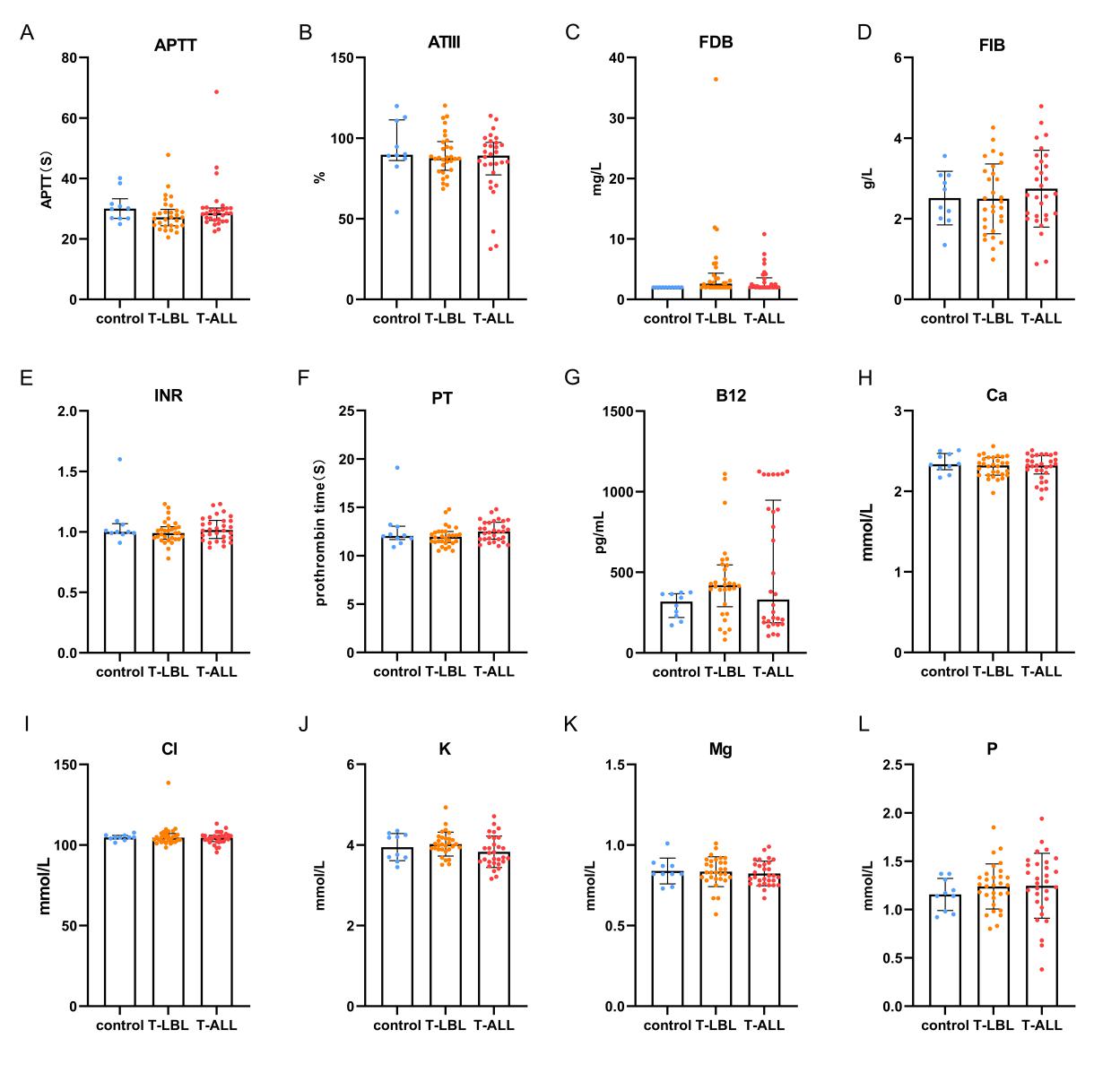


**Supplementary** **Figure 2.** Comparison of peripheral blood indicators of coagulation function, levels of trace elements, and other biochemical indices between the control, T-LBL, and T-ALL groups


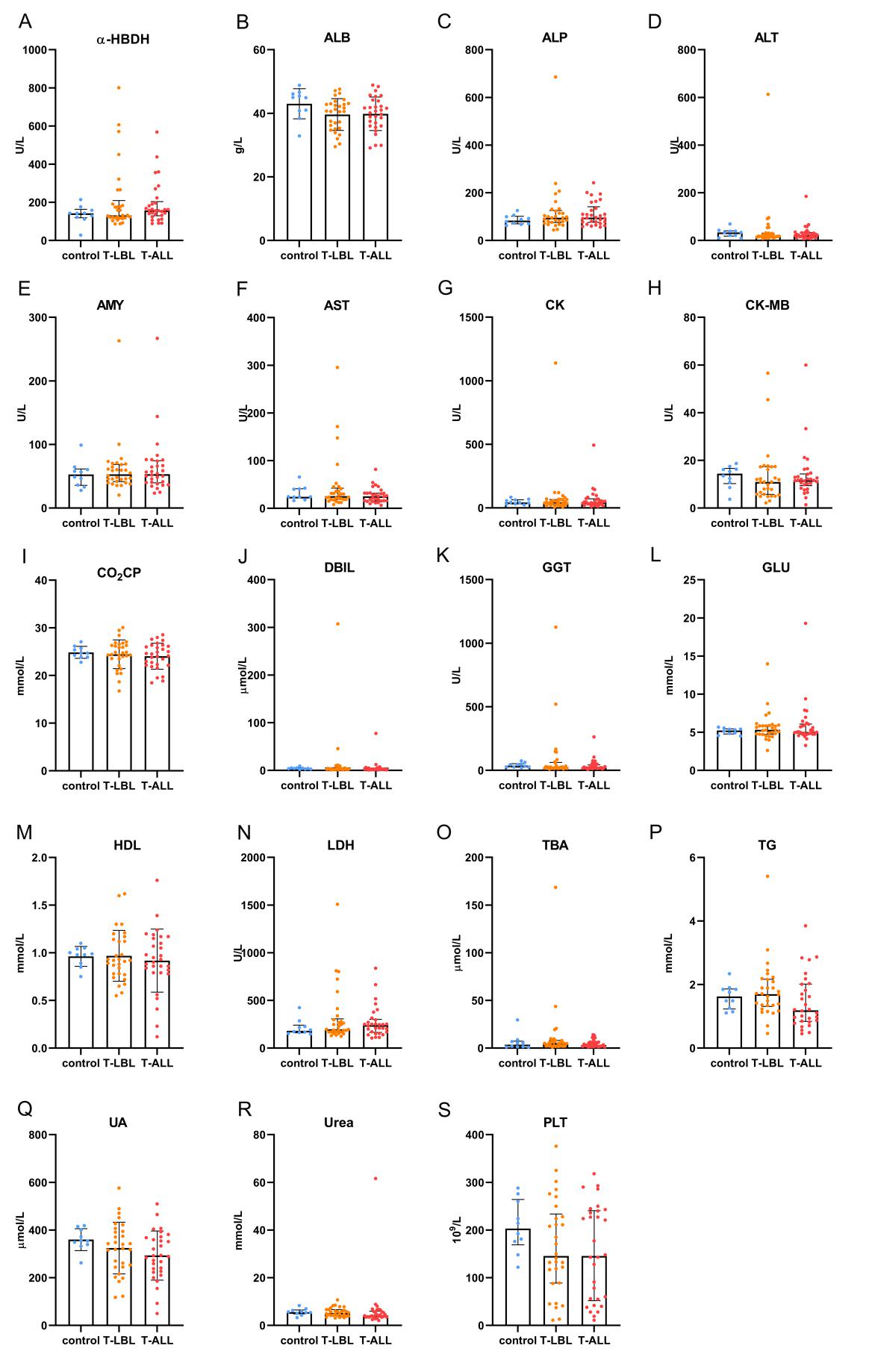


**Supplementary** **Figure 3.** Comparison of clinical and biochemical data on peripheral blood indices of liver and kidney function, blood lipids, and platelets between the control, T-LBL, and T-ALL groups

**Supplementary** **Table 1.** Comparison of clinical data between the T-LBL, T-ALL, and control groups

|  | Control | T-LBL | T-ALL | *P* value |
| --- | --- | --- | --- | --- |
| Sample number | 10 | 30 | 30 |  |
| Gender (male/female) | 7/3 | 21/9 | 23/30 | 0.825 |
| Age | 55.50(46.00-63.00) | 52.00(41.00-64.00) | 19.00(13.00-30.00) | ＜0.001 |
| WBC | 5.17(3.78-6.83) | 5.29(4.02-8.64) | 2.87(1.77-5.88) | 0.005 |
| PLT | 203.00(176.00-260.00) | 145.50(55.00-240.00) | 145.50(55.00-240.00) | 0.326 |
| HBG | 143.40±16.03 | 115.50±25.96 | 109.40±28.06 | 0.002 |
| NEUT% | 69.58±10.69 | 47.12±24.92 | 53.57±20.70 | 0.010 |
| NEUT# | 3.34(2.36-5.03) | 2.52(1.73-4.22) | 1.35(0.73-3.62) | 0.010 |
| LYMPH% | 23.00±8.88 | 36.81±22.48 | 35.37±20.40 | 0.168 |
| LYMPH# | 1.05(0.81-1.33) | 1.41(0.81-1.33) | 0.93(0.60-1.35) | 0.024 |
| TP | 71.66±6.00 | 68.10±9.80 | 63.51±8.41 | 0.024 |
| ALB | 42.99±4.73 | 39.62±4.99 | 39.84±5.29 | 0.178 |
| GLB | 28.10(26.00-31.80) | 26.20(23.60-29.20) | 24.50(20.00-26.60) | 0.005 |
| ALT | 33.35(20.90-39.20) | 18.30(15.50-30.50) | 23.00(15.20-32.80) | 0.561 |
| AST | 24.20(23.70-40.70) | 25.65(18.50-41.10) | 25.17(15.90-31.70) | 0.538 |
| ALP | 83.50(70.50-99.90) | 95.30(76.40-123.60) | 95.60(77.70-134.80) | 0.490 |
| GGT | 38.10(25.00-48.90) | 25.20(17.80-61.30) | 19.70(16.50-41.00) | 0.186 |
| TBA | 3.62(1.36-6.82) | 5.04(2.25-7.98) | 3.31(2.09-6.33) | 0.338 |
| TBIL | 15.00(11.00-18.30) | 15.00(11.00-18.30) | 10.55(7.50-17.30) | 0.018 |
| DBIL | 4.05(3.10-5.20) | 2.85(2.20-4.90) | 2.30(1.50-4.40) | 0.067 |
| Urea | 5.56(5.05-6.32) | 5.23(4.13-6.65) | 4.19(3.48-5.75) | 0.138 |
| Cr | 69.30(60.00-70.40) | 62.35(50.00-83.00) | 52.50(38.10-62.60) | 0.040 |
| UA | 359.70±46.14 | 324.48±108.52 | 293.23±103.05 | 0.166 |
| LDH | 181.30(162.60-225.90) | 196.35(168.90-296.70) | 240.95(167.80-297.20) | 0.612 |
| CK | 42.55(32.10-62.50) | 46.10(25.90-66.10) | 45.45(28.70-70.80) | 0.986 |
| CK-MB | 14.40(10.80-16.30) | 10.95(5.80-17.30) | 11.65(9.80-14.20) | 0.354 |
| a-HBDH | 142.45(121.60-158.60) | 131.90(117.90-190.90) | 157.00(131.30-196.70) | 0.510 |
| AMY | 52.85(36.40-60.40) | 4.00(3.89-4.16) | 3.75(3.58-4.17) | 0.839 |
| K | 3.95±0.34 | 4.02±0.30 | 3.83±0.39 | 0.105 |
| Na | 140.85(140.2-142.50) | 138.60(137.40-140.80) | 138.95(135.98-140.43) | 0.020 |
| Cl | 104.75(104.10-106.00) | 104.65(102.40-107.10) | 104.45(102.20-106.00) | 0.945 |
| Ca | 2.34(2.29-2.46) | 2.32(2.20-2.42) | 2.32(2.23-2.44) | 0.623 |
| P | 1.16±0.17 | 1.24±0.23 | 1.25±0.34 | 0.650 |
| Mg | 0.84±0.08 | 0.84±0.09 | 0.82±0.08 | 0.825 |
| CO2CP | 24.85±1.26 | 24.45±3.01 | 24.04±2.73 | 0.683 |
| GLU | 5.24(4.85-5.41) | 5.32(4.76-6.04) | 5.04(4.76-6.04) | 0.798 |
| TG | 1.63(1.26-1.85) | 1.70(1.34-2.16) | 1.19(0.85-2.01) | 0.104 |
| CHO | 3.97(3.75-4.17) | 4.65(4.13-4.86) | 3.38(2.84-.74) | 0.012 |
| HDL | 0.96±0.10 | 0.97±0.27 | 0.92±0.33 | 0.770 |
| LDL | 2.16(2.12-2.57) | 2.85(2.68-3.03) | 2.23(1.55-2.96) | 0.002 |
| FA | 17.26(14.67-18.56) | 26.25(19.60-31.17) | 11.79(7.04-16.24) | 0.000 |
| B12 | 318.00(229.00-365.00) | 419.50(302.00-544.10) | 331.00(189.00-894.00) | 0.198 |
| F | 125.75(104.20-174.70) | 178.00(145.70-190.80) | 259.65(89.50-585.20) | 0.124 |
| EPO | 9.05(1.56-12.93) | 9.47(7.85-23.88) | 31.87(14.15-167.30) | 0.000 |
| Iron | 18.64(17.18-25.91) | 18.66(15.30-20.11) | 16.57(6.81-22.63) | 0.397 |
| UIBC | 2.51±0.67 | 33.11±9.36 | 30.54±16.57 | 0.497 |
| TIBC | 60.10(50.33-67.32) | 57.30(53.10-59.30) | 54.53(43.12-59.64) | 0.404 |
| ISAT | 0.33(0.27-0.51) | 0.29(0.22-0.32) | 0.28(0.15-0.49) | 0.308 |
| PT | 12.05(11.80-13.00) | 11.95(11.40-12.50) | 12.50(11.70-13.40) | 0.117 |
| INR | 1.00(0.99-1.06) | 0.99(0.94-1.04) | 1.02(0.95-1.09) | 0.543 |
| APTT | 30.00(26.80-31.60) | 27.15(24.40-29.40) | 28.50(26.20-30.20) | 0.168 |
| TT | 17.85(16.90-19.20) | 17.65(17.00-18.40) | 16.45(15.80-17.10) | 0.002 |
| FIB | 2.51±0.67 | 2.49±0.87 | 2.75±0.95 | 0.514 |
| ATIII | 89.70(87.40-110.80) | 87.60(80.30-97.60) | 89.15(78.70-97.10) | 0.568 |
| FDB | 2.00(2.00-2.00) | 2.60(2.00-4.00) | 2.10(2.00-3.40) | 0.206 |
| DD | 0.22(0.19-0.25) | 0.70(0.37-0.99) | 0.43(0.19-0.88) | 0.001 |
| IgG | 14.55(10.30-16.60) | 10.45(8.98-12.70) | 10.05(8.50-11.40) | 0.052 |
| IgA | 2.00(1.46-3.04) | 1.57(1.02-2.17) | 1.37(0.97-1.99) | 0.159 |
| IgM | 0.91(0.70-1.21) | 0.67(0.44-0.89) | 0.63(0.44-0.89) | 0.140 |
| C3 | 0.82±0.26 | 0.97±0.27 | 0.96±0.23 | 0.231 |
| C4 | 0.20(0.16-0.29) | 0.26(0.21-0.34) | 0.25(0.21-0.30) | 0.401 |
| CRP | 1.48(1.25-2.28) | 3.48(1.51-11.20) | 3.82(1.82-9.72) | 0.039 |
| RF | 20.00±0.00 | 44.53±93.48 | 20.00±0.00 | 0.263 |
| ASO | 36.50(28.50-48.20) | 31.35(26.40-46.10) | 45.50(23.80-74.90) | 0.318 |

Note: Normally distributed data are represented by mean ± standard deviation, while non-normally distributed data are represented by M (Q1-Q3).

**Supplementary** **Table 2.** Pairwise comparisons of clinical data between the T-LBL, T-ALL, and control groups

|  | *P* value |  |  |  |
| --- | --- | --- | --- | --- |
|  | Control vs T-LBL | Crontrol vs T-ALL | T-LBL vs T-ALL | Comparison between three groups |
| Age | 1.000 | ＜0.001 | ＜0.001 | ＜0.001 |
| WBC | 1.000 | 0.205 | 0.004 | 0.005 |
| PLT | - | - | - | 0.326 |
| HBG | 0.004 | 0.001 | 0.364 | 0.002 |
| NEUT% | 0.006 | 0.047 | 0.253 | 0.022 |
| NEUT# | 0.606 | 0.017 | 0.102 | 0.010 |
| LYMPH% | 0.026 | 0.035 | 0.992 | 0.168 |
| LYMPH# | 0.453 | 1.000 | 0.022 | 0.024 |
| TP | 0.271 | 0.013 | 0.047 | 0.024 |
| ALB | - | - | - | 0.178 |
| GLB | 0.845 | 0.014 | 0.039 | 0.005 |
| ALT | - | - | - | 0.561 |
| AST | - | - | - | 0.538 |
| ALP | - | - | - | 0.490 |
| GGT | - | - | - | 0.186 |
| TBA | - | - | - | 0.338 |
| TBIL | 0.383 | 0.020 | 0.275 | 0.018 |
| DBIL | - | - | - | 0.067 |
| Urea | - | - | - | 0.138 |
| Cr | 1.000 | 0.325 | 0.048 | 0.040 |
| UA | - | - | - | 0.166 |
| LDH | - | - | - | 0.612 |
| CK | - | - | - | 0.986 |
| CK-MB | - | - | - | 0.354 |
| a-HBDH | - | - | - | 0.510 |
| AMY | - | - | - | 0.839 |
| K | - | - | - | 0.105 |
| Na | 0.036 | 0.021 | 1.000 | 0.020 |
| Cl | - | - | - | 0.945 |
| Ca | - | - | - | 0.623 |
| P | - | - | - | 0.105 |
| Mg | - | - | - | 0.825 |
| CO2CP | - | - | - | 0.683 |
| GLU | - | - | - | 0.798 |
| TG | - | - | - | 0.104 |
| CHO | 0.225 | 1.000 | 0.013 | 0.012 |
| HDL | - | - | - | 0.770 |
| LDL | 0.032 | 1.000 | 0.005 | 0.002 |
| FA | 0.166 | 0.501 | ＜0.001 | ＜0.001 |
| B12 | - | - | - | 0.198 |
| F | - | - | - | 0.124 |
| EPO | 0.827 | 0.001 | 0.001 | ＜0.001 |
| Iron | - | - | - | 0.397 |
| UIBC | 0.879 | 0.626 | 0.845 | 0.497 |
| TIBC | - | - | - | 0.404 |
| ISAT | - | - | - | 0.308 |
| PT | - | - | - | 0.117 |
| INR | - | - | - | 0.543 |
| APTT | - | - | - | 0.168 |
| TT | 0.703 | 0.008 | 0.001 | 0.002 |
| FIB | - | - | - | 0.514 |
| ATIII | - | - | - | 0.330 |
| FDB | 0.111 | 0.495 | 0.194 | 0.206 |
| DD | 0.001 | 0.048 | 0.246 | 0.001 |
| IgG | - | - | - | 0.052 |
| IgA | - | - | - | 0.159 |
| IgM | - | - | - | 0.140 |
| C3 | - | - | - | 0.231 |
| C4 | - | - | - | 0.401 |
| CRP | 0.064 | 0.044 | 1.000 | 0.039 |
| RF | 0.410 | - | 0.410 | 0.263 |
| ASO | - | - | - | 0.318 |

Note: The absolute values of PLT, ALT, AST, ALP, GGT, TBA, DBIL, Urea, LDH, CK, CK-MB, a-HBDH, AMY, Cl, Ca, GLU, TG, B12, F, Iron, TIBC, ISAT, PT, INR, APTT, IgG, IgA, IgM, C4, and ASO do not conform to the normal distribution, and significant differences between samples were not detected using non-parametric tests; thus, multiple comparisons were not performed. Single-factor analysis of variance did not detect statistically significant differences between samples for LYMPH%, ALB, UA, K, P, Mg, CO2CP, HDL, UIBC, FIB, ATIII, C3, and RF; thus, no post-hoc comparisons were performed.

**Supplementary Table S3.** Multiparameter analysis of clinical data in T-LBL, T-ALL, and control groups with VIP values greater than 1

| Rank | Original variable | VIP value |
| --- | --- | --- |
| 1 | F | 4.60924 |
| 2 | B12 | 3.64972 |
| 3 | LDH | 2.33206 |
| 4 | a-HBDH | 1.81929 |
| 5 | GGT | 1.81874 |
| 6 | EPO | 1.36563 |
| 7 | FA | 1.36413 |
| 8 | UA | 1.23417 |
| 9 | ALP | 1.14825 |

**Supplementary Table 4.** The top five clinical indicators showing the highest correlation coefficients in the T-LBL, T-ALL, and control groups, shown by multi-parameter analysis

| Rank | Original variable | VIP value |
| --- | --- | --- |
| 1 | HBG | 0.410021 |
| 2 | TIBC | 0.393007 |
| 3 | UA | 0.384629 |
| 4 | LDL | 0.355762 |
| 5 | TT | 0.351014 |

**Supplementary Table 5.** DEGs of control vs T-ALL identified in GSE48558

| Gene symbol (up-regulated DEGs) |
| --- |
| NELL2, LRRN3, TC2N, ABCD2, VSIG1, GIMAP4, TGFBR3, GZMK, GPR15, RASGRF2, LDLRAP1, CCR7, ZBTB38, TRABD2A, SAMD3, PIK3IP1, ZBTB38, SAMHD1, KLRG1, CTLA4, JAML, TSHZ2, KLRK1, CCL5, MID2, GPR171, SLFN12L, ANK3, PATJ, MYBL1, GBP5, AK5, GBP2, DPP4, IL6ST, IL10RA, RNF157, GZMA, IKZF3, TIGIT, GIMAP5, TRAV8-3, CYTIP, RORA, SESN3, GIMAP7, GBP2, IPCEF1, GPR155, TMEM204, DSC1, PCED1B, HCP5, CD226, ITK, MAML2, CD8B, BTLA, APP, SCARNA17, HLA-F, FCRL6, NR3C2, THEMIS, CLEC2D, INPP4B, PRF1, KLRD1, TRAJ17, ABCB1, GNLY, PIM2, LOC100131541, KIAA1324, CD27, IL7R, IL18RAP, KLRB1 |

**Supplementary Table 6.** DEGs of control vs T-ALL identified in GSE48558

| Gene symbol (down-regulated DEGs) |
| --- |
| RAB32, CCDC88A, CDCA2, CDK2AP1, FAM72A, MEF2C, STIL, TFPI, GINS1, RNASE2, EXO1, CEP55, SPC25, SCFV, AADAT, CHEK1, CENPI, PLK4, XRCC2, CD1E, WEE1, GPSM2, GCSAML, NETO1, CCNE2, CDC45, FOXM1, CDCA7, GAB2, CDK1, SPRED2, TUSC3, CCNA2, BTBD3, CDH2, GUCY1B3, E2F7, ARHGAP11A, SGO1, IGHM, ANKRD50, ADGRE2, KIF14, KIF23, HIST1H2BK, KIF11, RAD51AP1, LOC102723407, CCNB2, KIAA0101, SOX4, STMN1, KIAA0226L, SCFV, HIST2H3D, OVOS, HIST2H3D, CD34, UHRF1, KCNK17, MIR181B1, SYK, LPCAT2, TTK, BUB1, GRK3, CNTLN, LRP12, CENPU, HIST1H1B, SCN3A, CENPF, PRC1, PRR11, CKS2, FAM111B, SPRY2, KIF15, SORT1, SCFV, NEIL3, KIF20A, HHIP, PXDN, CPXM1, PTPRD, NCAPG, ESCO2, DLGAP5, ASPM, MKI67, FAT1, RBBP8, ANLN, TPX2, CKAP2L, GXYLT2, BUB1B, SKA3, KNL1, FADS1, MZB1, CD1B, TOP2A, ARPP21, BCL11A, MED12L, TCF4, SCFV, UGT3A2, GNB4, ZNF730, MYB, NUSAP1, IGLJ3, P2RX1, FADS2, HIST1H3B, DTL, B4GALT6, TYMS, DAPK1, GUCY1A3, DNTT, KIAA0101, NDST3, ERG, GNA15, NREP, TSPAN7 |

**Supplementary Table 7.** Control vs T-ALL: the top five most significant Gene Ontology enrichments

| Category | Term | Count | % | PValue | Genes |
| --- | --- | --- | --- | --- | --- |
| Biological Process (BP) | cell division | 23 | 12.10526316 | 5.33E-12 | GPSM2, CDCA2, CDCA7, KIF14, NCAPG, BUB1B, KIF11, KNL1, SKA3, SGO1, CCNA2, ASPM, CCNB2, TPX2, CENPF, WEE1, CCNE2, PRC1, RBBP8, CKS2, CDK1, BUB1, SPC25 |
|  | chromosome segregation | 10 | 5.263157895 | 6.96E-08 | TOP2A, SGO1, CENPF, CDCA2, TTK, ESCO2, SKA3, BUB1, DLGAP5, SPC25 |
|  | mitotic cell cycle | 10 | 5.263157895 | 9.00E-06 | PLK4, TPX2, CENPF, WEE1, XRCC2, MYB, KIF11, SKA3, MYBL1, KIF15 |
|  | G2/M transition of mitotic cell cycle | 7 | 3.684210526 | 1.31E-05 | CCNA2, APP, WEE1, ABCB1, CHEK1, CDK1, FOXM1 |
|  | mitotic spindle organization | 7 | 3.684210526 | 1.31E-05 | GPSM2, STIL, STMN1, TTK, KIF11, DLGAP5, SPC25 |
| Cellular Component (CC) | centrosome | 21 | 11.05263158 | 1.40E-07 | PLK4, GPSM2, STIL, CKAP2L, XRCC2, KIF23, AK5, SKA3, KIF15, SGO1, ASPM, CCNB2, CCDC88A, CENPF, CDC45, CCNE2, CHEK1, CDK1, DTL, CEP55, CNTLN |
|  | spindle | 11 | 5.789473684 | 7.56E-07 | ASPM, TPX2, CENPF, PRC1, NUSAP1, BUB1B, KIF23, TTK, KIF20A, KIF11, KIF15 |
|  | external side of plasma membrane | 17 | 8.947368421 | 5.06E-06 | IGHM, FCRL6, CD1E, HLA-F, CD1B, TGFBR3, KLRK1, P2RX1, CD27, CTLA4, CD226, KLRD1, CCR7, IL6ST, IL7R, CLEC2D, CD34 |
|  | cell surface | 20 | 10.52631579 | 5.18E-06 | IGHM, APP, ABCB1, KLRB1, SORT1, HHIP, ANK3, TFPI, HLA-F, CD1B, TGFBR3, DPP4, KLRK1, CDH2, CD8B, PXDN, CD226, CCR7, TIGIT, CLEC2D |
|  | kinetochore | 10 | 5.263157895 | 1.29E-05 | SGO1, CENPU, CENPF, CENPI, BUB1B, TTK, KNL1, SKA3, BUB1, SPC25 |
| Molecular Function (MF) | microtubule binding | 12 | 6.315789474 | 3.21E-05 | CCDC88A, TPX2, CENPF, PRC1, NUSAP1, KIF14, KIF23, KIF20A, KIF11, MID2, DLGAP5, KIF15 |
|  | protein kinase binding | 16 | 8.421052632 | 9.21E-05 | SYK, BCL11A, KIF14, KIF11, FOXM1, CCNA2, SPRED2, TPX2, CDH2, CCNE2, PRC1, CKS2, SPRY2, CD226, KIF20A, CNTLN |
|  | protein homodimerization activity | 18 | 9.473684211 | 4.24E-04 | TOP2A, GBP5, APP, ABCD2, BCL11A, AADAT, ZBTB38, GZMA, TYMS, IKZF3, GIMAP7, MID2, DPP4, CCDC88A, CENPF, JAML, CCL5, GBP2 |
|  | coreceptor activity | 5 | 2.631578947 | 0.00103294 | TGFBR3, GPR15, IL18RAP, CD8B, IL6ST |
|  | protein binding | 135 | 71.05263158 | 0.001333808 | APP, PATJ, MAML2, HHIP, PRF1, BUB1B, RORA, GIMAP5, MKI67, IKZF3, GIMAP7, NR3C2, IL18RAP, SESN3, VSIG1, CDH2, STMN1, CHEK1, MYB, NUSAP1, PIM2, SOX4, GBP5, MEF2C, DAPK1, ZBTB38, THEMIS, ANK3, ESCO2, KNL1, HLA-F, RNASE2, IPCEF1, SGO1, RAB32, INPP4B, CD8B, CCNE2, CD226, ERG, KIF20A, IL6ST, DSC1, ABCB1, KLRB1, TSHZ2, CDCA7, NCAPG, NREP, SKA3, CD1B, MED12L, DPP4, RAD51AP1, CCNB2, GRK3, KLRK1, BTBD3, TSPAN7, BTLA, PLK4, STIL, GPR15, XRCC2, FAM111B, PTPRD, TPX2, FAT1, GNB4, CDK1, CD27, TCF4, KLRD1, IL7R, LRP12, TOP2A, ITK, GPSM2, ABCD2, KIF14, KIF11, FOXM1, KIF15, SPRED2, ADGRE2, GNLY, EXO1, CTLA4, LDLRAP1, CYTIP, CD34, DLGAP5, CEP55, SYK, FCRL6, KIF23, DNTT, GAB2, CCNA2, TGFBR3, GCSAML, MZB1, CKS2, DTL, FAM72A, UHRF1, TTK, SAMHD1, CDC45, CCL5, PCED1B, RBBP8, GBP2, TIGIT, ANKRD50, BUB1, E2F7, SAMD3, KLRG1, CENPU, SORT1, IL10RA, GZMA, NELL2, GZMK, CENPF, WEE1, PIK3IP1, PRC1, CENPI, P2RX1, LPCAT2, SPRY2, CDK2AP1, SPC25 |
| Kyoto Encyclopedia of Genes and Genomes Pathway  (KEGG) | Cell cycle | 13 | 6.842105263 | 2.23E-07 | BUB1B, TTK, ESCO2, KNL1, SGO1, CCNA2, CCNB2, WEE1, CDC45, CCNE2, CHEK1, CDK1, BUB1 |
|  | Hematopoietic cell lineage | 7 | 3.684210526 | 9.72E-04 | LOC102723407, CD8B, CD1E, DNTT, IL7R, CD34, CD1B |
|  | Cell adhesion molecules | 8 | 4.210526316 | 0.002227093 | PTPRD, CDH2, CD8B, CTLA4, CD226, TIGIT, HLA-F, CD34 |
|  | Cellular senescence | 7 | 3.684210526 | 0.009309155 | CCNA2, CCNB2, CCNE2, CHEK1, CDK1, FOXM1, HLA-F |
|  | p53 signaling pathway | 5 | 2.631578947 | 0.01047387 | CCNB2, SESN3, CCNE2, CHEK1, CDK1 |

**Supplementary Table 8.** Control vs T-ALL: The top five most significant Gene Ontology enrichments for upregulated DEGs

| Category | Term | Count | % | PValue | Genes |
| --- | --- | --- | --- | --- | --- |
| Biological Process (BP) | immune response | 11 | 14.86486486 | 5.60E-06 | TGFBR3, IL18RAP, CD8B, CCL5, GZMA, CTLA4, CCR7, SAMHD1, IL7R, GBP2, HLA-F |
|  | positive regulation of natural killer cell mediated cytotoxicity | 4 | 5.405405405 | 9.89E-05 | KLRK1, IL18RAP, KLRD1, CD226 |
|  | cellular response to cytokine stimulus | 4 | 5.405405405 | 3.00E-04 | GBP5, CCR7, LDLRAP1, GBP2 |
|  | negative regulation of T cell apoptotic process | 3 | 4.054054054 | 4.88E-04 | CCL5, CD27, IL7R |
|  | cell surface receptor signaling pathway | 7 | 9.459459459 | 7.33E-04 | KLRB1, FCRL6, CD27, KLRD1, IL7R, CLEC2D, KLRG1 |
| Cellular Component (CC) | plasma membrane | 39 | 52.7027027 | 2.32E-07 | APP, ITK, PATJ, ABCB1, KLRB1, RASGRF2, PRF1, SAMHD1, DPP4, RNF157, KLRK1, IL18RAP, VSIG1, TMEM204, GPR171, BTLA, CTLA4, CCR7, LDLRAP1, TIGIT, KLRG1, GPR15, FCRL6, IL10RA, ANK3, IPCEF1, HLA-F, TGFBR3, CD8B, JAML, PIK3IP1, CD27, CD226, KLRD1, IL6ST, IL7R, CLEC2D, DSC1, TRABD2A |
|  | external side of plasma membrane | 12 | 16.21621622 | 4.91E-07 | TGFBR3, KLRK1, FCRL6, CTLA4, CD27, KLRD1, CCR7, CD226, IL6ST, IL7R, HLA-F, CLEC2D |
|  | cell surface | 13 | 17.56756757 | 1.58E-06 | APP, ABCB1, KLRB1, ANK3, HLA-F, TGFBR3, DPP4, KLRK1, CD8B, CD226, CCR7, TIGIT, CLEC2D |
|  | integral component of membrane | 35 | 47.2972973 | 3.21E-05 | APP, ABCD2, ABCB1, KLRB1, PRF1, GIMAP5, SCARNA17, DPP4, KLRK1, VSIG1, TMEM204, GPR171, CTLA4, CCR7, TIGIT, GPR155, SLFN12L, KLRG1, GPR15, LRRN3, IL10RA, HLA-F, TGFBR3, NELL2, INPP4B, CD8B, JAML, PIK3IP1, CD226, KLRD1, IL6ST, IL7R, CLEC2D, DSC1, TRABD2A |
|  | receptor complex | 6 | 8.108108108 | 8.52E-04 | TGFBR3, APP, CD8B, KLRD1, IL6ST, NR3C2 |
| Molecular Function (MF) | coreceptor activity | 5 | 6.756756757 | 2.24E-05 | TGFBR3, GPR15, IL18RAP, CD8B, IL6ST |
|  | protein homodimerization activity | 12 | 16.21621622 | 3.20E-05 | DPP4, GBP5, APP, ABCD2, JAML, CCL5, ZBTB38, GZMA, GBP2, IKZF3, GIMAP7, MID2 |
|  | protein binding | 54 | 72.97297297 | 0.003269248 | APP, ITK, PATJ, ABCD2, MAML2, PRF1, RORA, GIMAP5, IKZF3, GIMAP7, NR3C2, IL18RAP, SESN3, VSIG1, GNLY, CTLA4, PIM2, LDLRAP1, CYTIP, GBP5, FCRL6, ZBTB38, THEMIS, ANK3, IPCEF1, HLA-F, TGFBR3, INPP4B, CD8B, CD226, IL6ST, DSC1, ABCB1, KLRB1, TSHZ2, SAMHD1, DPP4, KLRK1, CCL5, PCED1B, BTLA, TIGIT, GBP2, SAMD3, KLRG1, GPR15, IL10RA, GZMA, NELL2, GZMK, PIK3IP1, CD27, KLRD1, IL7R |
|  | transmembrane signaling receptor activity | 5 | 6.756756757 | 0.003752747 | KLRB1, FCRL6, CD27, KLRD1, CLEC2D |
|  | identical protein binding | 14 | 18.91891892 | 0.004072287 | GBP5, APP, PRF1, SAMHD1, IKZF3, GIMAP7, MID2, DPP4, KLRK1, CCL5, CD226, TIGIT, GBP2, IL6ST |
| Kyoto Encyclopedia of Genes and Genomes Pathway  (KEGG) | Viral protein interaction with cytokine and cytokine receptor | 5 | 6.756756757 | 7.25E-04 | IL18RAP, CCL5, IL10RA, CCR7, IL6ST |
|  | Cytokine-cytokine receptor interaction | 7 | 9.459459459 | 0.001218744 | IL18RAP, CCL5, IL10RA, CD27, CCR7, IL6ST, IL7R |
|  | Cell adhesion molecules | 5 | 6.756756757 | 0.003811337 | CD8B, CTLA4, CD226, TIGIT, HLA-F |
|  | Graft-versus-host disease | 3 | 4.054054054 | 0.012892662 | PRF1, KLRD1, HLA-F |
|  | Autoimmune thyroid disease | 3 | 4.054054054 | 0.020058127 | PRF1, CTLA4, HLA-F |

**Supplementary Table 9.** Control vs T-ALL: the top five most significant Gene Ontology enrichments for downregulated DEGs

| Category | Term | Count | % | PValue | Genes |
| --- | --- | --- | --- | --- | --- |
| Biological Process (BP) | cell division | 23 | 19.82758621 | 1.82E-16 | GPSM2, CDCA2, CDCA7, KIF14, NCAPG, BUB1B, KIF11, KNL1, SKA3, SGO1, CCNA2, ASPM, CCNB2, TPX2, CENPF, WEE1, CCNE2, PRC1, RBBP8, CKS2, CDK1, BUB1, SPC25 |
|  | chromosome segregation | 10 | 8.620689655 | 1.05E-09 | TOP2A, SGO1, CENPF, CDCA2, TTK, ESCO2, SKA3, BUB1, DLGAP5, SPC25 |
|  | mitotic spindle organization | 7 | 6.034482759 | 8.24E-07 | GPSM2, STIL, STMN1, TTK, KIF11, DLGAP5, SPC25 |
|  | mitotic cell cycle | 9 | 7.75862069 | 2.03E-06 | PLK4, TPX2, CENPF, WEE1, XRCC2, MYB, KIF11, SKA3, KIF15 |
|  | mitotic spindle assembly checkpoint | 5 | 4.310344828 | 2.89E-05 | CENPF, BUB1B, TTK, BUB1, SPC25 |
| Cellular Component (CC) | centrosome | 20 | 17.24137931 | 1.82E-10 | PLK4, GPSM2, STIL, CKAP2L, XRCC2, KIF23, SKA3, KIF15, SGO1, ASPM, CCNB2, CCDC88A, CENPF, CDC45, CCNE2, CHEK1, CDK1, DTL, CEP55, CNTLN |
|  | spindle | 11 | 9.482758621 | 7.40E-09 | ASPM, TPX2, CENPF, PRC1, NUSAP1, BUB1B, KIF23, TTK, KIF20A, KIF11, KIF15 |
|  | nucleus | 60 | 51.72413793 | 1.09E-07 | ARHGAP11A, TOP2A, HHIP, KIF14, BUB1B, KIF11, FOXM1, MKI67, EXO1, CHEK1, MYB, NUSAP1, SOX4, DLGAP5, MEF2C, SYK, DAPK1, KIF23, DNTT, ESCO2, KNL1, CCNA2, ASPM, NEIL3, CCNE2, KIF20A, ERG, DTL, PRR11, CDCA2, UHRF1, CDCA7, NCAPG, TTK, NREP, TYMS, RAD51AP1, CCNB2, CDC45, BTBD3, RBBP8, BUB1, E2F7, GINS1, PLK4, CENPU, BCL11A, FAM111B, TPX2, CENPF, WEE1, CENPI, PRC1, FAT1, CDK1, SPRY2, CDK2AP1, TCF4, ZNF730, SPC25 |
|  | kinetochore | 10 | 8.620689655 | 2.20E-07 | SGO1, CENPU, CENPF, CENPI, BUB1B, TTK, KNL1, SKA3, BUB1, SPC25 |
|  | midbody | 9 | 7.75862069 | 7.83E-06 | ASPM, ANLN, CENPF, PRC1, CDK1, KIF14, KIF23, KIF20A, CEP55 |
| Molecular Function (MF) | protein kinase binding | 15 | 12.93103448 | 1.59E-06 | SYK, BCL11A, KIF14, KIF11, FOXM1, CCNA2, SPRED2, TPX2, CDH2, CCNE2, PRC1, CKS2, SPRY2, KIF20A, CNTLN |
|  | microtubule binding | 11 | 9.482758621 | 2.72E-06 | CCDC88A, TPX2, CENPF, PRC1, NUSAP1, KIF14, KIF23, KIF20A, KIF11, DLGAP5, KIF15 |
|  | microtubule motor activity | 5 | 4.310344828 | 3.81E-04 | KIF14, KIF23, KIF20A, KIF11, KIF15 |
|  | ATP binding | 19 | 16.37931034 | 0.002700457 | PLK4, TOP2A, SYK, DAPK1, XRCC2, KIF14, BUB1B, TTK, KIF23, KIF11, MKI67, KIF15, WEE1, GRK3, P2RX1, CHEK1, CDK1, KIF20A, BUB1 |
|  | protein serine/threonine/tyrosine kinase activity | 9 | 7.75862069 | 0.003700332 | PLK4, WEE1, SYK, DAPK1, CHEK1, CDK1, BUB1B, TTK, BUB1 |
| Kyoto Encyclopedia of Genes and Genomes Pathway  (KEGG) | Cell cycle | 13 | 11.20689655 | 1.06E-09 | BUB1B, TTK, ESCO2, KNL1, SGO1, CCNA2, CCNB2, WEE1, CDC45, CCNE2, CHEK1, CDK1, BUB1 |
|  | Cellular senescence | 6 | 5.172413793 | 0.005541835 | CCNA2, CCNB2, CCNE2, CHEK1, CDK1, FOXM1 |
|  | Hematopoietic cell lineage | 5 | 4.310344828 | 0.005846116 | LOC102723407, CD1E, DNTT, CD34, CD1B |
|  | Oocyte meiosis | 5 | 4.310344828 | 0.015305477 | SGO1, CCNB2, CCNE2, CDK1, BUB1 |
|  | p53 signaling pathway | 4 | 3.448275862 | 0.016778026 | CCNB2, CCNE2, CHEK1, CDK1 |

**Supplementary Table 10.** DEGs showing the top 10 highest network degree values in the PPI networks

| Node | CDK1 | CCNA2 | MKI67 | TOP2A | FOXM1 | EXO1 | KIF11 | CHEK1 | BUB1B | BUB1 |
| --- | --- | --- | --- | --- | --- | --- | --- | --- | --- | --- |
| Description | down | down | down | down | down | down | down | down | down | down |
| Degree | 122 | 120 | 112 | 110 | 110 | 110 | 108 | 106 | 106 | 104 |

**Supplementary Table 11.** Upregulated DEGs showing the top 10 highest network degree values in the PPI networks

| Node | GZMA | IL7R | GZMK | CCL5 | CCR7 | PRF1 | TIGIT | CTLA4 | KLRB1 | KLRD1 |
| --- | --- | --- | --- | --- | --- | --- | --- | --- | --- | --- |
| Description | up | up | up | up | up | up | up | up | up | up |
| Degree | 54 | 50 | 46 | 44 | 42 | 42 | 42 | 40 | 40 | 40 |

**Supplementary Table 12.** Downregulated DEGs showing the top 10 highest network degree values in the PPI networks

| Node | CCNA2 | CDK1 | KIF11 | FOXM1 | EXO1 | TOP2A | MKI67 | BUB1B | CHEK1 | BUB1 |
| --- | --- | --- | --- | --- | --- | --- | --- | --- | --- | --- |
| Description | down | down | down | down | down | down | down | down | down | down |
| Degree | 118 | 118 | 108 | 108 | 108 | 108 | 106 | 106 | 104 | 104 |
